# Mapping Antibody Domain Exposure on Nanoparticle Surfaces Using DNA-PAINT

**DOI:** 10.1021/acsnano.3c02195

**Published:** 2023-06-07

**Authors:** Marrit M. E. Tholen, Bas J. H. M. Rosier, Robin T. Vermathen, Céline A. N. Sewnath, Cornelis Storm, Laura Woythe, Cristina Izquierdo-Lozano, Roger Riera, Marjolein van Egmond, Maarten Merkx, Lorenzo Albertazzi

**Affiliations:** † Department of Biomedical Engineering, Institute for Complex Molecular Systems (ICMS), Eindhoven University of Technology, 5612 AZ Eindhoven, The Netherlands; ‡ Molecular Cell Biology and Immunology, Amsterdam UMC, Vrije Universiteit Amsterdam, De Boelelaan 1117, 1081 HV Amsterdam, The Netherlands; § Cancer Biology and Immunology, Cancer Center Amsterdam, 1081 HV Amsterdam, The Netherlands; ∥ Cancer Immunology, Amsterdam Institute for Infection and Immunity, 1081 HV Amsterdam, The Netherlands; ⊥ Department of Applied Physics, Institute for Complex Molecular Systems (ICMS), Eindhoven University of Technology, 5612 AZ Eindhoven, The Netherlands; # Department of Surgery, Amsterdam UMC, Vrije Universtiteit Amsterdam, De Boelelaan 1117, 1081 HV Amsterdam, The Netherlands

**Keywords:** super-resolution microscopy, DNA-PAINT, nanomedicine, nanoparticles, heterogeneity, antibodies

## Abstract

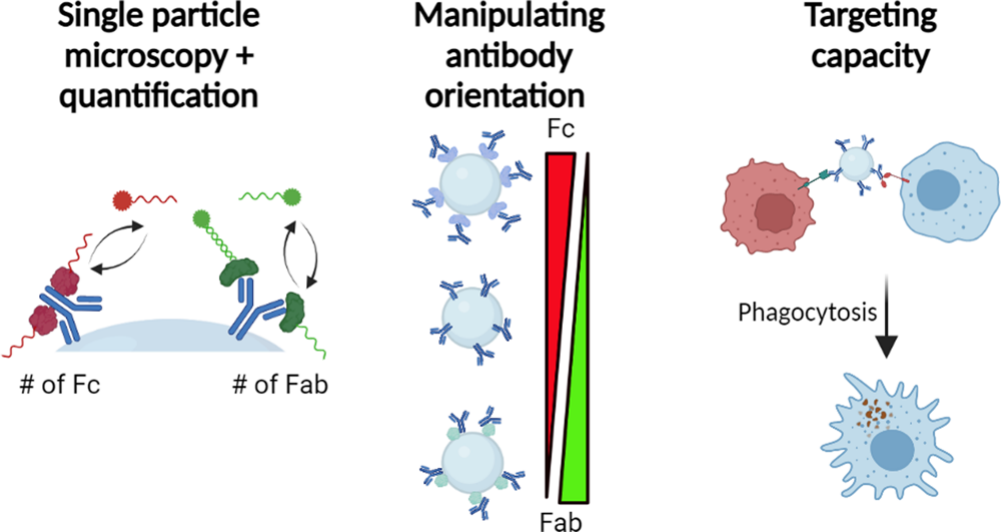

Decorating nanoparticles with antibodies (Ab) is a key strategy for targeted drug delivery and imaging. For this purpose, the orientation of the antibody on the nanoparticle is crucial to maximize fragment antibody-binding (Fab) exposure and thus antigen binding. Moreover, the exposure of the fragment crystallizable (Fc) domain may lead to the engagement of immune cells through one of the Fc receptors. Therefore, the choice of the chemistry for nanoparticle-antibody conjugation is key for the biological performance, and methods have been developed for orientation-selective functionalization. Despite the importance of this issue, there is a lack of direct methods to quantify the antibodies’ orientation on the nanoparticle’s surface. Here, we present a generic methodology that enables for multiplexed, simultaneous imaging of both Fab and Fc exposure on the surface of nanoparticles, based on super-resolution microscopy. Fab-specific Protein M and Fc-specific Protein G probes were conjugated to single stranded DNAs and two-color DNA-PAINT imaging was performed. Hereby, we quantitatively addressed the number of sites per particle and highlight the heterogeneity in the Ab orientation and compared the results with a geometrical computational model to validate data interpretation. Moreover, super-resolution microscopy can resolve particle size, allowing the study of how particle dimensions affect antibody coverage. We show that different conjugation strategies modulate the Fab and Fc exposure which can be tuned depending on the application of choice. Finally, we explored the biomedical importance of antibody domain exposure in antibody dependent cell mediated phagocytosis (ADCP). This method can be used universally to characterize antibody-conjugated nanoparticles, improving the understanding of relationships between structure and targeting capacities in targeted nanomedicine.

## Introduction

Antibodies (Ab) are ubiquitous in therapy and diagnosis, both *in vivo* and *in vitro*, due to their high specificity and affinity toward molecular targets. In the emerging field of nanomedicine, antibodies are often immobilized on the surface of nanoparticles (NPs) to promote targeting selectivity toward a specific cell population.^1−6^ Additionally, by presenting multiple antibodies on a small surface area, NP multivalent targeting can be achieved, which increases binding and uptake compared to monovalent binding.^2^ For instance, the anticancer efficacy of trastuzumab and anti-PD-L1 antibodies was increased when they were immobilized on NPs.^7,8^ Although this illustrates the potential of antibody-covered NPs in biomedical applications, a convincing clinical application has so far not been demonstrated.

One of the main underlying bottlenecks is that individual NP characteristics, such as the number, availability, and functionality of the antibodies on the NP surface, are difficult to characterize^9,10^ and therefore the design and development of optimal targeted particles is far from trivial. Parameters such as intra- and interparticle variations, antibody inactivation due to unfolding or crowding, and incorrect antibody orientation are often overlooked but greatly influence the efficacy of the particle to target receptors *in vivo*.^2^ Control over the exposure of the fragment antibody-binding (Fab) or the fragment crystallizable (Fc) region of an antibody immobilized on NPs is a key parameter for the targeting.^3−5,11^ In the case of active targeting, the Fab domain is involved in recognizing the receptor or target of interest, while the Fc domain is mostly involved in recognition by the immune system, but it can also be recognized by Fc receptors on the surface of epithelial and endothelial cells.^7,12−18^ Therefore, the antibody orientation and the resulting exposure of Fab domains, Fc domains, or combinations of the two is crucial for targeting.

Antibody availability and orientation on NPs are strongly dependent on the conjugation strategy used. Although many strategies are available, coupling to native amino acid residues in the antibody, based on *N*-hydroxysulfosuccinimide (NHS) or maleimide chemistry, is still most widely used, but results in a lack of site specificity and therefore poor control over antibody orientation.^3,19−21^ Strategies that provide more control over the orientation of the antibody to the surface of particles have been developed, e.g., by site-directed modification of the Fc domain, through antibody recognition by specific proteins (e.g., protein G that binds to the Fc region of some antibodies), or by conjugation through glycan remodeling,^7,20−26^ among others.

Although several methods for assessing the antibody to NP ratio have been developed, e.g., the addition of fluorescently labeled or radioactive ligands, these approaches only give an ensemble average and do not discriminate between Fc- and Fab-exposing antibodies.^3,6,23^ Single-particle techniques can provide the necessary resolution to overcome these limitations. Recently, transmission electron microscopy (TEM), flow cytometry and super-resolution microscopy (SRM) techniques have been developed to quantify and map epitopes on the surface of NPs.^27−29^ Recent advances in the quantification of antibodies on the surface of NPs with an SRM technique called DNA Points Accumulation for Imaging in Nanoscale Topography (DNA-PAINT) enabled researchers to characterize NPs both geometrically and functionally with high sensitivity and precision. This technique relies on the transient binding and unbinding between complementary docking and fluorophore-labeled imager DNA strands, allowing for nanoscale resolution (10–20 nm).^29−31^ By using quantitative PAINT (qPAINT), in which the kinetics of the DNA sequences are used to convert number of localizations into number of molecules, the exact number of functional ligands can be quantified.^32,33^ Compared to other microscopy techniques, photobleaching is negligible, and multiplexing can be achieved by using multiple DNA sequences.^34−36^ For example, our lab studied the spatial distribution and heterogeneity of Fab domains of antibody-functionalized polystyrene NPs by using DNA labeled antigens. However, these probes could only be applied to the Fab domain of one type of antibody.^29^ These reports show the potential of super-resolution microscopy to characterize Ab-functionalized NPs but has two main limitations: (i) they do not provide information about Ab orientation, and (ii) they require a specific probe for every antibody, lacking generality.

Here, we introduce a pair of imaging probes that recognize the Fc and the Fab region of an antibody and are generally applicable to a wide range of antibodies to quantify their orientation. The probes consist of either the Fc-targeting protein G (pG) or the Fab-targeting protein M (pM) The pG domain is a 9.6-kDa protein, derived from Streptococcal bacteria, that is naturally able to bind to the C_H_2–C_H_3 junction of a broad range of antibodies with high affinity (*K*_d_ = 10 nM).^37−40^ The pM protein (∼40 kDa) was discovered and characterized in 2014 by Grover et al. and originates from *Mycoplasma genitalium*. It has a large binding domain, principally binding to the highly conserved κ and λ domains of a Fab fragment with a *K*_d_ in the low nanomolar range (1–5 nM).^41^ By coupling these proteins to single stranded DNAs, these proteins can be used for multiplexed DNA-PAINT to gain more insight into the antibody’s orientation and the efficacy of the NP conjugation method. Moreover, these proteins also have potential to be used for directed orientation of antibodies on the surface of particles. First, the method was validated for quantification of antibody orientation on NPs at a single-particle level. Additionally, by comparing different sizes of nanoparticles and multiple orientation strategies, the effect of changing these parameters on the antibody orientation was shown and, most importantly, interparticle heterogeneity could be studied. To robustly interpret the super-resolution data in terms of Ab orientation a theoretical model was developed and used to fit the obtained results based on geometrical considerations. Lastly, to show the biomedical relevance of antibody orientation and correlate our microscopy results with a biological function, the ability of nanoparticles to trigger antibody-dependent cell-mediated phagocytosis (ADCP) was explored, showing that random conjugation of antibodies could be the most beneficial for immune therapy. The presented method for single-particle quantification of antibody orientation is generally applicable and provides a tool to understand relationships between structure and targeting capacities in targeted nanomedicine.

## Results and Discussion

### Methodology

**Figure 1:**
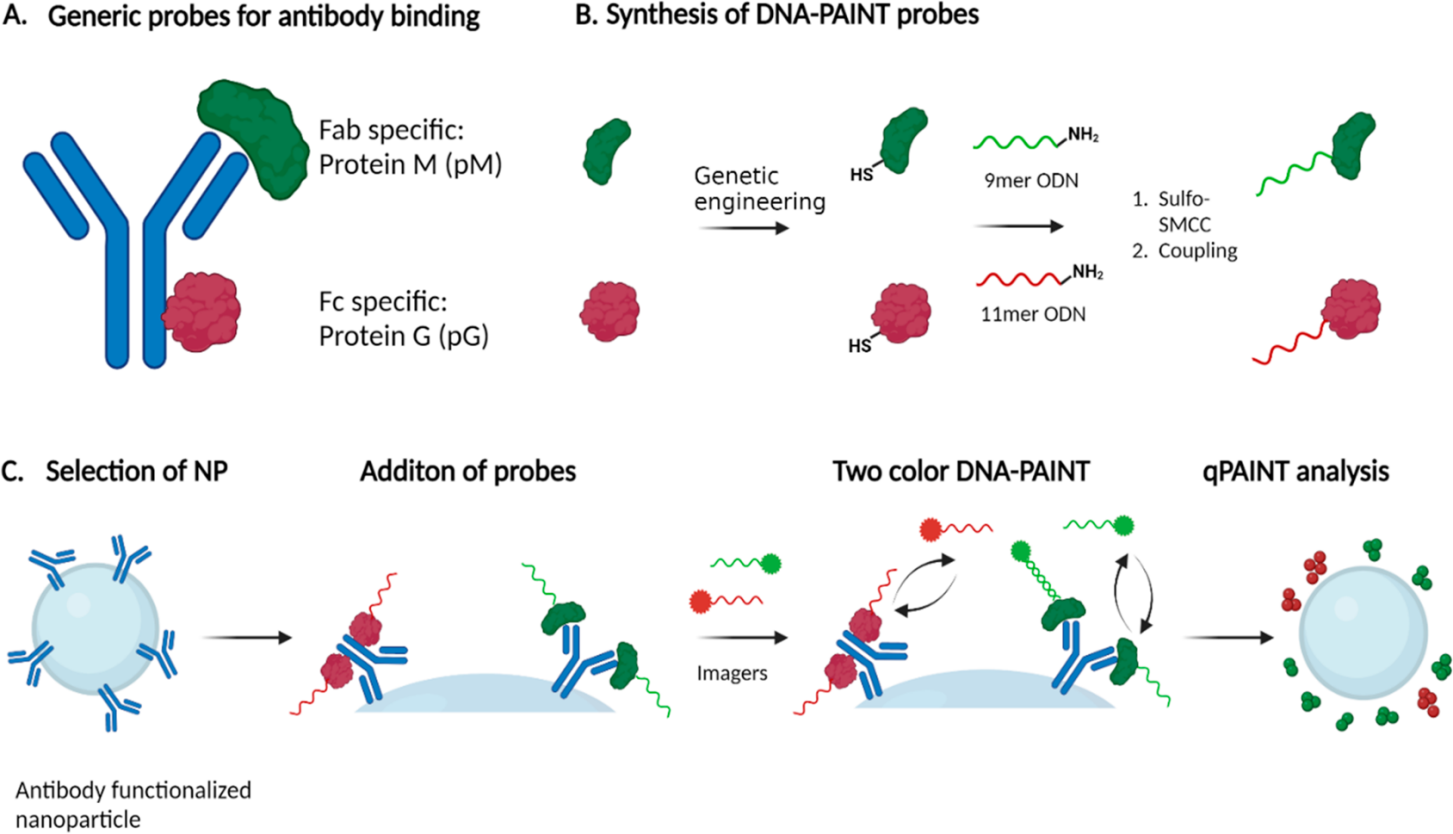
Schematic overview of the strategy for the mapping of antibodies on the surface of nanoparticles using DNA-PAINT. Schematics are not drawn to scale. (A) Schematic representation of the binding sites of pM and pG, used to map the Fab and Fc domains, respectively. (B) Engineering of proteins to develop DNA-PAINT compatible probes. First, the proteins are genetically engineered to express a site-specifically inserted cysteine-handle. Thereafter, the two different ODNs, which were activated by the bifunctional cross-linker SMCC, are coupled to the proteins, which results in the probes. (C) The nanoparticles functionalized with antibodies are incubated with the probes, with pG and pM specifically binding to the fragment crystallizable (Fc) region and the antibody-binding fragment (Fab) region, respectively. Then, the imagers for DNA-PAINT are added, with a specific imager for each probe. Simultaneous imaging is started, and using qPAINT, the amount of antibodies and their orientation is quantified. Schematic was created with BioRender.com.

Our generally applicable method for quantification of antibody orientation on NPs is outlined in Figure 1 and extensively discussed in the Experimental Section. To enable simultaneous quantification of both the Fc and Fab antibody domains, two-color DNA-PAINT with orthogonal docking strands coupled to pG and pM were developed. Well-defined conjugation of a single short DNA strand to the protein probes is an essential feature for DNA-PAINT.^42^ To achieve this, we employed a benchtop-compatible strategy for site-specific conjugation of oligonucleotides to antibody-binding adapter proteins, which was developed by Cremers et al. (2019) and shown to be applicable for DNA-PAINT.^37,43,44^ A single cysteine residue was inserted into both proteins such that it is not located in the vicinity of the antibody-binding interface. Amino-modified oligodeoxynucleotides (ODNs) were activated using succinimidyl-4-(*N*-maleimidomethyl)cyclohexane-1-carboxylate (SMCC), after which a 5-fold excess was used for coupling to pG and pM with yields >90%. Purification was performed using strep-column affinity chromatography for removal of excess ODNs and anion-exchange chromatography for removal of unreacted protein after which pure pG-ODN and pM-ODN probes were obtained (Figure 1B, Figure S1).^37,43^

In a typical imaging experiment used throughout this study, particles were synthesized, incubated with probes and—after washing away unreacted probes—imaged with DNA-PAINT for the quantification of antibodies (Figure 1C). The probes specifically bind to distinct sites on an antibody (two Fc and two Fab domains, for pG and pM, respectively) and, therefore, will only bind to the nanoparticle if the respective domain is exposed to the solution and can contribute to target binding. For DNA-PAINT imaging, NPs incubated with both probes were adsorbed onto the glass of an imaging chamber. Then, the complementary imager strands, i.e., Cy3B and Atto-647N labeled ODNs, were added to the solution. Transient binding of the short fluorescently labeled imager strands (7 and 9 nucleotides, respectively) with the docking strands on the surface of the NPs through DNA hybridization allowed for single-molecule localization. The selected imager strands were optimized for their length and kinetics previously.^31^ After image analysis, a two-color image, based on the localization of individual binding events, containing information about the distribution and number of both antibody domains was obtained, which was analyzed with qPAINT for quantification.^29,30^

### Characterization of NPs with Random Orientation

Our first experiment aims to demonstrate the feasibility and accuracy of our two-color imaging approach to map the Fc and Fab domains of antibodies immobilized on the surface of NPs. A therapeutic antibody targeting the epidermal growth factor receptor (cetuximab, Ctx) was conjugated to NPs following a two-step reaction, that was expected to result in random orientation. As previously described,^18^ the surface of carboxylic acid-activated silica NPs was activated using *N*-(3-(dimethylamino)propyl)-*N*′-ethylcarbodiimide hydrochloride (EDC), followed by conjugation of the primary amines of Ctx to the particles.

As a proof of concept, we investigated the specificity between the respective docking and imager strands. To this end, Ctx-functionalized 300 nm particles were incubated with either the pG or pM probe, after which both imagers in optimized concentrations were added during imaging. Signal from only one color in the presence of only one probe and a colocalizing signal from both colors in the case when both probes are present is expected to be observed.

**Figure 2:**
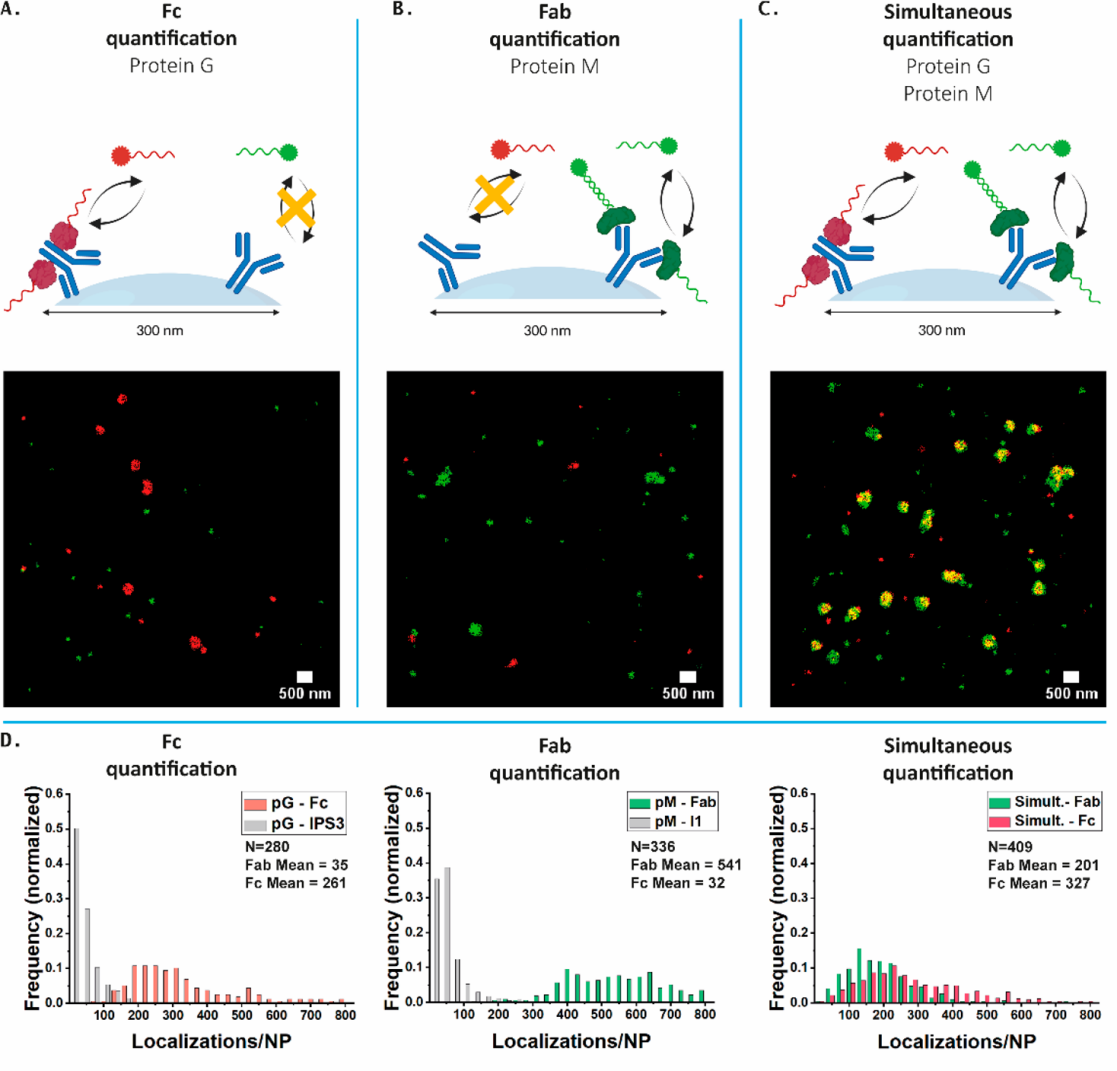
Randomly oriented cetuximab on the surface of silica nanoparticles. (A) DNA-PAINT super-resolution image obtained using pG-ODN with both imagers present in solution (10,000 frames, 11 Hz). The specific imager (red) solely binds to the particles, whereas the noncomplementary imager (green) mainly has aspecific interactions. (B) DNA-PAINT super-resolution image obtained using pM-ODN with both imagers present in solution (10,000 frames, 11 Hz). The specific imager (green) binds specifically to the particles. On the contrary, the noncomplementary imager (red) mainly has aspecific interactions. (C) DNA-PAINT super resolution image obtained using both pG-ODN and pM-ODN and both imagers present in solution (10,000 frames, 11 Hz). Both imagers show specific interaction with the particles. (D) Histograms of the localizations/NP. The specificity that was observed in (B) is also reflected here. The gray bars represent background signal caused from aspecific binding of imager PS3 (IPS3) or imager 1 (I1). Simult.: simultaneous imaging in both green and red channel. N: number of particles analyzed. Fab mean: mean number of localizations in green channel. Fc mean: mean number of localizations in red channel. Scale bars: 500 nm. Schematic was created with BioRender.com.

As can be found in Figure 2A and B and Figure S5, for both pG-I1 (depicted in red) and pM-IPS3 (depicted in green), only the correct imager bound to the particles, giving a single-molecule localization per binding event, while the noncomplementary imager, IPS3 and I1 for pG and pM, respectively, only displayed negligible nonspecific interactions with the glass. During simultaneous imaging (Figure 2C and S2–S4), where both probes and both imagers were added, the signal of the probes colocalize, indicating specific binding to the particles. Analysis of the number of localizations per NP showed that nonspecific binding was low and there was a large interparticle heterogeneity (Figure 2D). Furthermore, batch-to-batch variation caused differences in the number of localizations, as can be seen by the difference in the number of localizations between the samples that were used for single and simultaneous imaging. These results combined show that our method allows for simultaneous mapping of the orientation of antibodies on the surface of the NPs.

**Figure 3:**
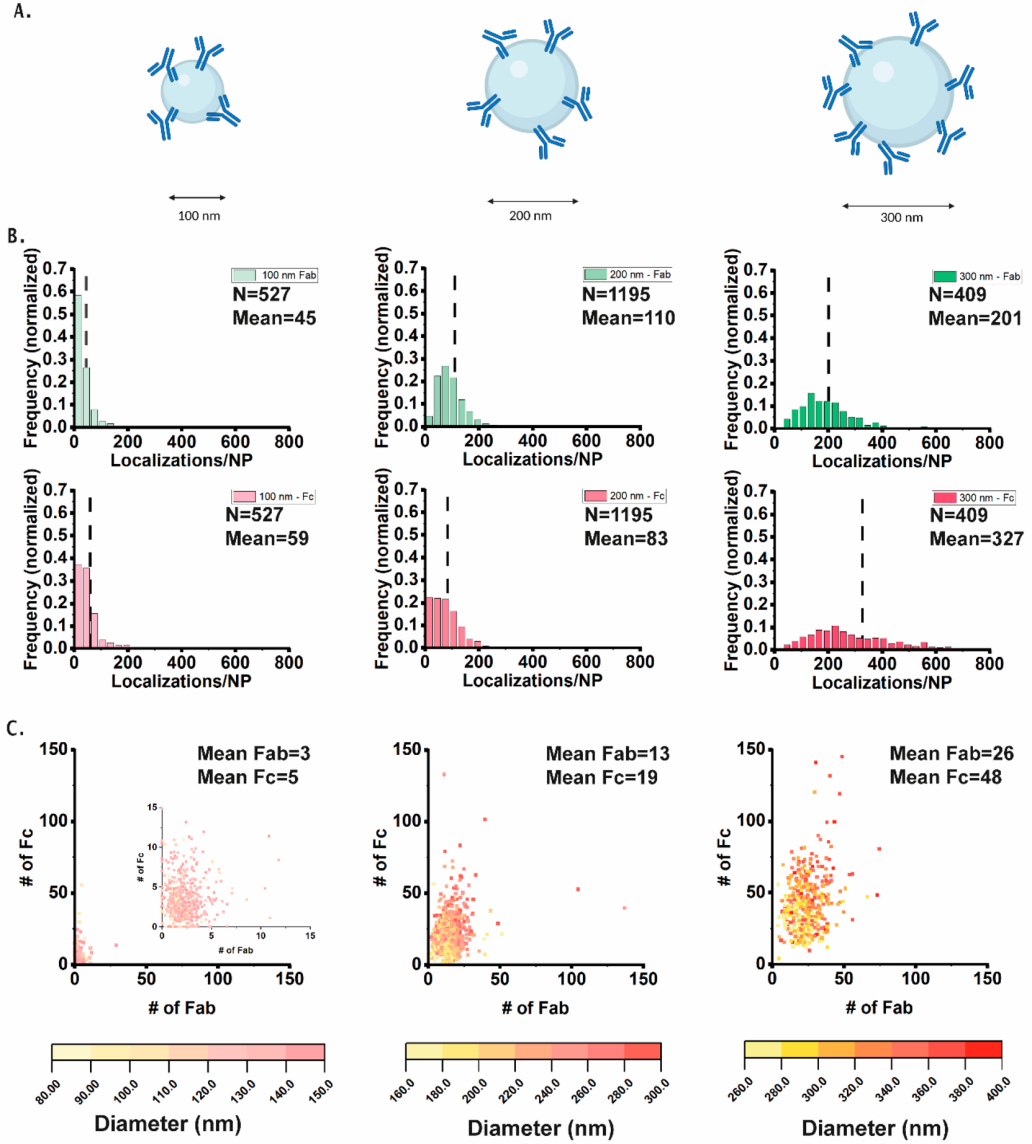
(A) Schematic representation of the different sizes of NPs. (B) Normalized frequency histograms of DNA-PAINT localizations per NP for 100, 200, and 300 nm randomly conjugated NPs. Graphs include the number of NPs analyzed (*N*) and the mean number of localizations per NP (Mean). Bin widths = 20. (C) Number of Fc per NP plotted over number of Fab per NP.

Next, we quantitatively assessed the effect of particle size on the distribution of accessible Fc and Fab domains of randomly immobilized antibodies (Figure 3). For this, NPs with different diameters ranging from 100 to 300 nm were chosen, while the conjugation incubation time, buffer pH, imaging conditions and the concentration of probes and imagers were kept constant. The chosen particles have similar characteristics in terms of surface charge (Figure S6), although the 300 nm particles have a charge density of 2 μmol/g compared to 1 μmol/g for the 100 and 200 nm particles according to the manufacturer. The histograms in Figure 3B represent the localization distribution of Fc and Fab domains for each NP size. Figure 3C highlights the corresponding number of Fab and Fc domains per individual NP, which is derived from the localizations based on the kinetic information on the imager and the mean dark time, as described in previous research and in the Experimental Section.^29,33^ The data for the 300 nm particles depicted in Figure 3B is the same as in Figure 2, but depicted in a different manner. The actual diameter of every NP was determined from our images and visualized as a color map in Figure 3C, while in Figure S7, the number of Fc or Fab domains is plotted as a function of the actual NP size. As expected, the number of localizations for both probes and therefore the number of both domains increased gradually with an increase in NP diameter due to the higher chance of reaction to a larger surface. Interestingly, while all NPs depicted a broad distribution of antibody domains, while, interestingly, the width of the distribution broadened with particle size (Figure 3C and Figure S7). This reflects a high level of interparticle heterogeneity, with larger-diameter NPs exhibiting a larger heterogeneity in the exposure of the immobilized antibodies, which is further reflected in the standard deviation (SD, Figure S8–S9 and Table S1). This is further confirmed by the coefficient of variation (CV), which was calculated to determine the interparticle heterogeneity. All NPs depict a broad distribution of antibody domains, with CVs ranging from 40 to 110%, while the surface areas corresponding to the calculated sizes, have CVs around 30% (Figure 3C, S7 and S8, Table S1). As nonsymmetrical distributions were observed, this indicated that the stochastic process of conjugation of antibodies to the surface is intertwined with different parameters like size and differences in carboxyl group distribution on the NP surface. It was shown before that the concentration of functional groups, reaction times, and conditions play a role.^6,32^ In future research, applying this method to a wide variety of particle types, charges, number of functional groups and conjugation strategies (e.g., varying the pH) would be valuable. In case of the particle size, the CVs range between 8 and 16%, and together with previous results, it can be concluded that the commercial particles have a rather homogeneous size distribution, suggesting that the conjugation plays a major role in this case.^18^ Together, these results show that our method based on multiplexed mapping of individual antibody domains using DNA-PAINT provides quantitative information about antibody density, accessibility, and sample heterogeneity.

### Characterization of NPs with Directed Orientation

In nanomedicine applications, it is often desired to preferentially target specific cell types with either the Fab or Fc domain of the antibody, for example to target a specific cell receptor expressed in the disease state (by Fab domains) or elicit an immune response (by Fc domains). Since randomly oriented antibodies on NPs were shown to have a slight preference for Fc exposure, as shown in Figure 3, which might lead to unwanted side-effects, it is valuable to develop an NP synthesis method using site-directed conjugation in order to gain control over antibody orientation on the NP surface. In previous studies, oriented immobilization on NPs using the Fc-binding protein G has been used to demonstrate that antibody orientation influences cellular uptake, although a quantitative analysis of the NPs themselves was lacking.^20,45^ We therefore tested the ability of our super-resolution method to find a difference between different site-specific methods to conjugate antibodies to particles.

**Figure 4:**
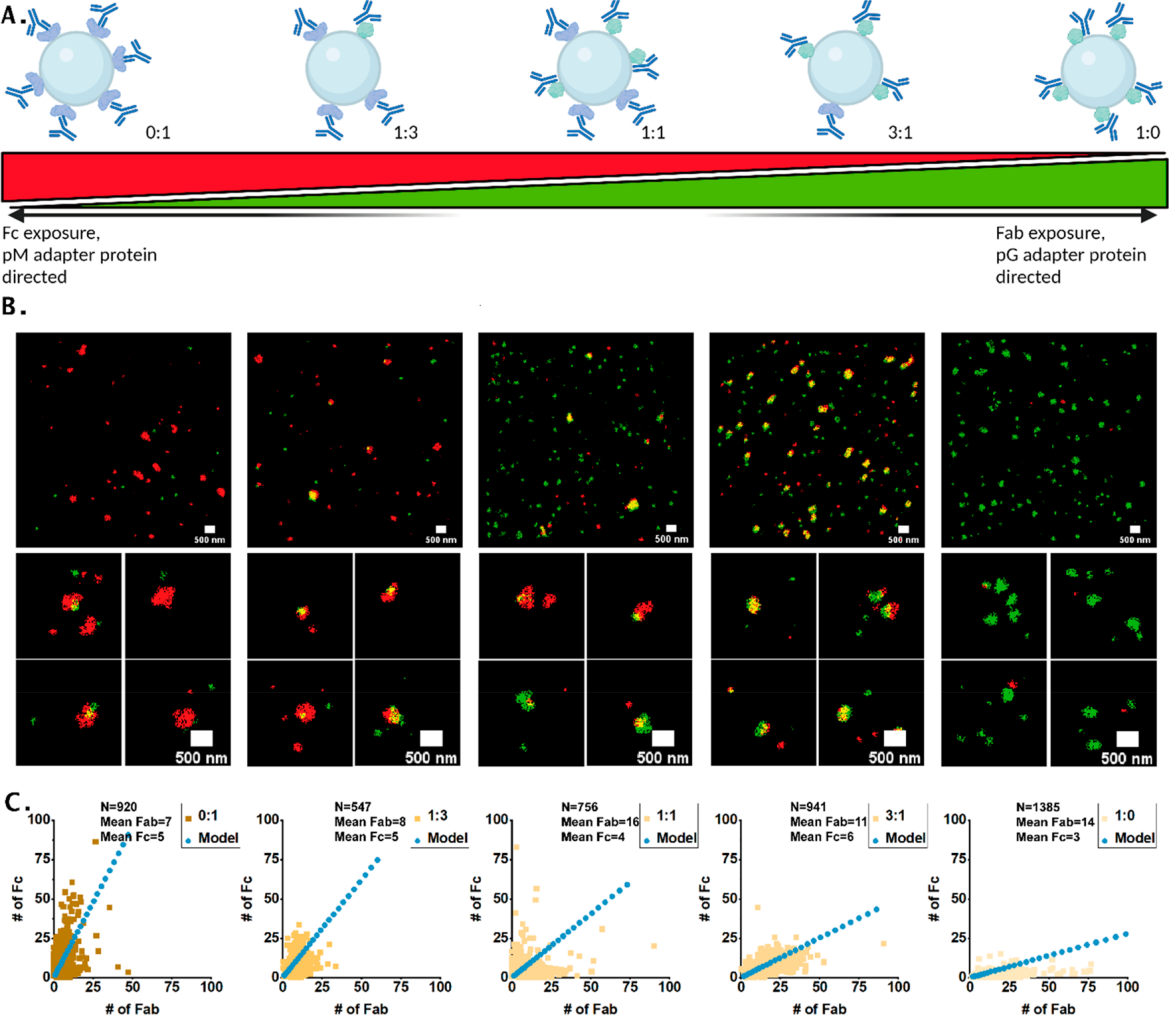
Manipulating the antibody exposure on silica nanoparticles by adapter proteins. (A) Schematic representation of the strategy to control the exposure of domains. Gradient represents the hypothesized ratio of Fc and Fab exposure. As extremes, particles functionalized with pM and pG are chosen to achieve full exposure of Fc and Fab domains, respectively. Three particles with a ratio of pG and pM coverage were chosen to show control. (B) Respective DNA-PAINT results of the particles with control over conjugation. As can be observed for the pM particles, Fc is dominantly exposed. In contrast, pG particles have a dominant exposure of Fab domains. The three intermediate particles have both domains exposed. (C) Scatter plots of the qPAINT results of the respective particles, calculated with use of the respective imager kinetics (Experimental Section). Blue line represents the results of the geometrical model, which predicts the Fab and Fc exposure. (Experimental Section). Scale bars: 500 nm. Schematics created with BioRender.com.

We hypothesized that the decoration of NPs with a well-defined mixture of antibody domain-binding pG and pM adapter proteins could enable the formation of different antibody domain exposure patterns (Figure 4A). Similar to the approach highlighted in Figure 1, pG and pM labeled with a single cysteine distant to the antibody-binding surface as the conjugation site were used, in order to direct antibody orientation on the NP surface. When antibodies bind to pM, the Fc domains will be exposed, while the Fab domains will be exposed when antibodies bind to pG. Quantitative characterization of the NPs with oriented antibodies can then be performed using the multiplexed mapping method with DNA-PAINT using pG-ODN and pM-ODN, as outlined in Figure 2. In short, 200 nm amino-functionalized silica NPs were activated using a heterobifunctional cross-linker (sulfosuccinimidyl 4-(*N*-maleimidomethyl) cyclohexane-1-carboxylate (Sulfo-SMCC)), after which pG and pM were reacted onto the particles. Subsequently, Ctx antibody was added to the particle mixture and incubated for 1 h to allow immobilization. To control the ratio of exposed Fab domains over Fc domains, five different NPs with a mix of pG and pM were prepared: 0:1, 1:3, 1:1, 3:1, and 1:0 (Figure 4A). In total, the proteins were added in a 1:1 molar ratio compared to the amount of NH_2_ groups on the particle.

Indeed, image analysis after DNA-PAINT acquisition revealed that the ratio between exposed Fc and Fab domains changed as a function of the ratio of pG and pM used in particle functionalization (Figure 4B, Figure S9–S11). When only the Fab-binding pM was used for antibody immobilization, Fc domains were predominantly detected (Figure 4B, left column), while the use of Fc-binding pG on the NPs resulted in exposure of the Fab domains of the immobilized antibodies (Figure 4B, right column). Importantly, the ratio between exposed Fc and Fab domains could be tuned by using a different ratio of pM:pG during NP preparation (Figure 4B, center columns). For the 0:1 particles, Fab exposure is still observed, which means that some Fab domains were still available with the pM conjugation, possibly due to the flexibility of the domains. Furthermore, these observations suggest different reaction yields of pG and pM during particle functionalization, which is in according with the different sizes of the proteins. When compared to the results found for 200 nm particles with random conjugation (Figure 3, center column), the overall number of both domains was lower, although an exception is observed for the Fab exposure in the 1:0 particles. This can be explained by a reduced number of available binding sites on the NP surface compared to EDC conjugation, as the surface area of the proteins used for orienting the antibodies is larger than EDC and there are thus less binding sites available. Furthermore, the surface charge of the bare particles differs from the carboxylic acid particles used for EDC conjugation, which might affect the conjugation (Figure S6). The standard deviation from the mean in the extreme 1:0 and 0:1 cases is smaller than the SD for the EDC conjugated NPs, suggesting a more homogeneous distribution of antibodies on the NPs (Figure S11, Table S2). It could be argued that one of the binding sites is already occupied for binding, potentially biasing the results, however, for each possible conjugation method, one domain is always blocked. For the intermediate cases the standard deviation from the mean slightly increased compared to the extremes, and for the Fab domains they were similar to the EDC-functionalized particles discussed above. Taken together, these results demonstrate that antibody orientation on the surface of NPs is possible by decorating particles with a mixture Fc- or Fab-binding adapter proteins before antibody immobilization. Multiplexed super-resolution imaging revealed that the ratio between exposed Fc and Fab domains can be rationally and gradually tuned, which enables exciting opportunities for the development of oriented antibody-NPs in preferential cellular uptake studies for various nanomedicine applications. This shows the potential of our method to compare and rank different types of chemistries for the control of Ab orientation on NPs.

### Validating the Results with a Computational Model

**Figure 5:**
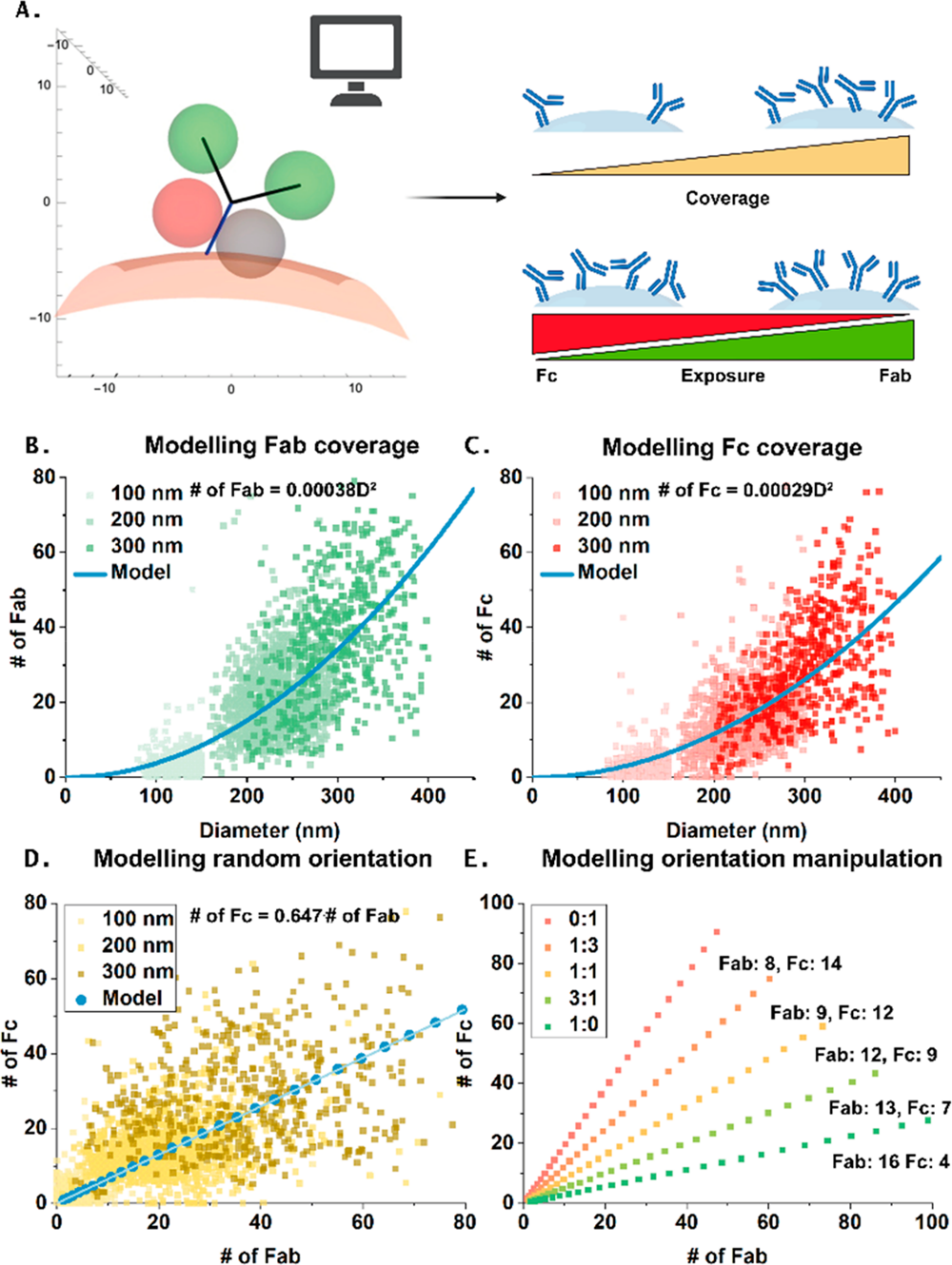
Computational model for verification of results. (A) Schematic representation. (B) Correlation between diameter and Fab coverage predicted by the model plotted on top of the data discussed in Figure 3. (C) Correlation between diameter and Fc coverage predicted by the model plotted on top of the data discussed in Figure 3. (D) Correlation between number of Fc and Fab as predicted by the model plotted on top of the data discussed in Figure 3. (E) Prediction of the model for the number of Fab and Fc in the orientation manipulated particles discussed in Figure 4. Schematic created with Biorender.com.

To better understand the factors that contribute to domain exposure in various conjugation scenarios and to better interpret the results, a computational model was developed that simulates the antibody orientation of the NPs in different conditions. This geometrical model has been used previously,^18^ and a detailed description of the model can be found in the Supporting Information (Table S3, Figure S12). A few assumptions were made: (1) The antibody was modeled as a Y-shaped molecule, with a single Fc stalk with a length of 6.5 nm and two Fab arms measuring 8 nm each with an opening angle of 100 degrees between them. The binding sites, for pG and pM are modeled as exclusion zones: spherical regions with a radius of 4 nm each, by taking the length of the DNA strands in account. Occlusion happens when any part of the exclusion zone is blocked by the particle surface (Figure 5A). (2) Every antibody has a 200 nm^2^ footprint. (3) Depending on the experiment, either a random deposition or an oriented deposition of cetuximab was assumed.

The first thing the model allows to compute by fitting the super-resolution data is the fraction of the area of the NP that is covered by antibodies, which results in a total functional surface accessibility of 4.2%. For the individual domains, the model showed that the exposure in absolute numbers correlates to area, as evidenced by a quadratic dependence on diameter (Figure 5B and C). The low areal fraction implies that the assumption can be made that neighboring antibodies do not interact or affect each other’s binding domain exposure, and that every orientation has an equal chance. Subsequently, the expected exposure of Fc and Fab domains were modeled. If conjugation were truly random, the model predicts 35% of antibodies have their Fc domain bound to the NP, and 65% bind with their Fab domain. These percentages can roughly be understood from the simple fact that the two Fab domains are further apart than the Fc domains. Important to note is that domain-binding statistics do not translate one-to-one into binding site exposure statistics; an Fc-bound configuration may still expose 0, 1, or 2 pG binding sites and, likewise, may occlude one or both pM binding sites. Averaging the availability of the four protein binding domains over all configurations, the random model predicted a slight predominance of Fc exposure as might be expected from the relative abundance of Fab-bound states: the ratio Fc:Fab is approximately 1.05 for random conjugation based on the stoichiometry of the antibody. However, as the light blue line in Figure 5D shows, the experimental Fc:Fab ratio was about 0.65. From this can be concluded that the orientation of the Ab is not fully random and there is, in fact, a small preference for Fc-binding which can account for the underestimation of the Fab-exposure. A possible explanation could be a higher accessibility of the lysine groups in the Fc domain to react with the EDC-activated particles. This tendency was included in the model by providing a small energetic advantage to Fc-binding, which shifted the natural ratio for Fc-Fab binding to 62%:38%. With this single correction, the observed binding and exposure ratios could accurately be captured. With these parameter settings, validated by experimental results, the effect of manipulating the antibody exposure, as was performed in Figure 4, were explored. Here, the model also qualitatively reproduced the experimental findings without further parameter adjustments, confirming the models accuracy. The model correctly displays that even though one of the domains is used for binding, the other one might still be available, which is depicted in the 0:1 and 1:0 cases, where the slopes of the model prediction do not reach 0 or infinity. Notably, the model supports the finding that there is a more likely orientation of the antibody due to the nonequal domain exposure in the 1:1 sample, supporting the hypothesis that there are other factors playing a role in the eventual orientation of the antibodies, which requires further investigation. Interestingly, very little dependence of binding site availability on NP radius was found, with only a weak effect at the smallest radii.

### Exploring the Effect of Orientation on Antibody Dependent Cell Mediated Phagocytosis (ADCP)

As a final part of this work, the ability of oriented nanoparticles to trigger ADCP and thereby killing of cancer cells by innate effector immune cells, was explored.^14^ This is instrumental in showing the correlation between super-resolution orientation analysis and biological functionality. ADCP is a particularly interesting phenomena as it requires both Fab and Fc exposure and the ratio of the two (i.e., Ab orientation) is crucial toward the biological efficacy. Moreover ADCP is a clinically relevant mechanism for monoclonal antibodies that can potentially be further enhanced by NP conjugation.

**Figure 6:**
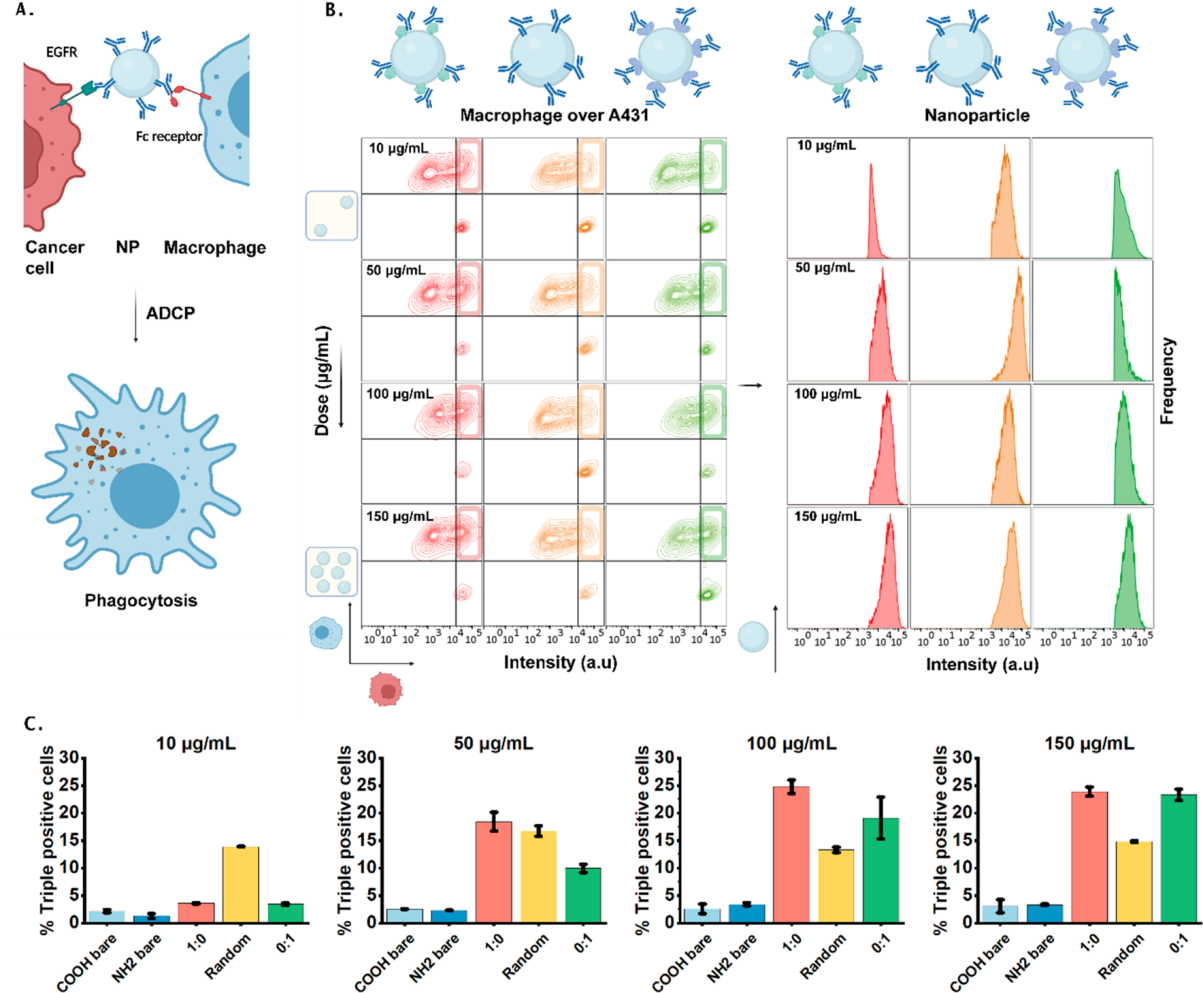
Antibody dependent cell mediated phagocytosis of nanoparticle triggered cells investigated by flow cytometry. (A) Schematic overview of the ADCP assay. A431 cells are spiked with nanoparticles (either 1:0, random or 0:1), harvested and cocultured with macrophages. (B) Phagocytosis is quantified using flow cytometry. First, phagocytosed cancer cells were selected by gating the A431 signal over the macrophage signal and selecting the double positive cells, indicated by the colored windows in the plot. Red: 1:0, orange: random, green: 0:1. Afterward, the amount of triple positive cells was determined by plotting the signal of the NPs within the double positive group and selecting the triple positive cells. (C) By dividing the number of triple positive cells over the total number of cells, the % of triple positive could be determined. As can be found, at a low dose the random particles trigger an effect; for the other particles, this happens at higher dosages. Schematics were created with Biorender.com.

First, a viability assay was performed using Alamar Blue to exclude NP toxicity. As was expected, the particles do not trigger noticeable cell death by themselves (Figure S13). The ability of the particles to engage the receptors on the nanoparticles, was assessed by incubating the nanoparticles with A431 EGFR overexpressing human squamous carcinoma cells and macrophages prepared from human peripheral blood mononuclear cells (PBMCs) individually. After incubation, bioaccumulation was assessed with flow cytometry. From these results it can be found that at the lowest dosage, the random particles are binding to the A431 cells at a higher level than the other particles (Figure S14B,C). For the higher dosages, the engagement of the receptor is similar. In case of the macrophages, engagement of the receptor is similar for all particles and dosages. To explore the ability of the particles to trigger ADCP, A431labeled with eFluor 670 following the manufacturers protocol, were exposed to the fluorescently labeled particles, either the 1:0 pG, 0:1 pM oriented particles or the 200 nm random particles, and cocultured with 3-octadecyl-2-[3-(3-octadecyl-2(3*H*)-benzoxazolylidene)-1-propenyl]-perchlorate (DiO) labeled macrophages. After incubation, uptake of nanoparticles by the cells was analyzed using flow cytometry. By plotting the signal in the red channel (eF670) over the signal in the blue channel (DiO), macrophages that are double positive for both DiO and eFluor 670 were identified, which is indicative of phagocytosis (Table S4) as fragments of “red” cancer cells are found inside the “green” macrophages. Furthermore, single eFluor 670 positive events reflect nonphagocytosed cancer cells, which were present in all conditions. These data allows to quantify ADCP as well as cancer cell killing. To obtain the amount of macrophages that not only have signal for the cancer cells, but also nanoparticles, the number of triple positive cells was calculated (Figure 6B and C and S15) by filtering for the double positive (red–blue) cells and plotting the signal in the yellow NP channel. From the signal in the yellow channel a slight dose dependency can be observed for the pG and pM functionalized NPs. For the EDC functionalized NPs, the signal seems to have an optimum around a dose of 50 μg/mL. However, to quantify this, the percentage of phagocytosis was calculated by dividing the number of triple positive cells over the total number of cells (Figure 6C). Clearly, all the antibody labeled particles trigger phagocytosis, while the control particles induce a low background phagocytosis. Strikingly, the randomly oriented particles trigger phagocytosis already at a significantly lower dose (10 μg/mL), while the pG 1:0 and pM 0:1 particles give a significant effect only with a dose higher than 50 μg/mL, displaying even stronger interaction than the randomly oriented ones. Combining both the results from the individual cell bioaccumulation assay and the ADCP assay, this supports the idea that for ADCP random orientation of antibodies is preferred as these NPs can engage both receptors more efficiently. At high concentrations also oriented particles are effective because these particles still expose a part of the other domain,, as reported in Figure 4, and the particles are still able to bind to both cell types. Furthermore, these results indicate that cellular uptake does not require a high number of antibodies on the surface of the NPs as the functional accessibility was only 4.2% as calculated by the computational model, which was also reported previously.^46^ For future research, it would be interesting to further explore the influence of antibody density and orientation on the ability to trigger ADCP.

## Conclusions

For active targeting using antibodies, it is crucial to have quantitative information on the antibody domain exposure and accessibility on the NP surface. Previously, this could only be done in bulk,^6^ or in case of single-particle imaging, only for Fab domains of specific antibodies,^29^ resulting in only a partial elucidation of the nanoparticle functionalities. In this work, we introduce a generic method for the quantification of both Fc and Fab antibody domains on the surface of various NPs. By using pG-ODN and pM-ODN with two distinct DNA docking strands, simultaneous multiplex mapping of both Fab and Fc domains can be achieved. Gaining knowledge about the number of both Fab and Fc domains on a developed material, this will result in more insight in the number of functional groups of the material as Fab and Fc have different targets in the body. DNA-PAINT allowed for quantitative insights into the inter- and intraparticle distribution of available domains. We were able to control the exposure of Fab and Fc domains by site-selective conjugation of antibodies to the NP surface. All of these findings were supported by a simple geometric model, which reveals that even EDC conjugation is not completely random, helping the interpretation of the data. In an ADCP assay, all particle formulations triggered ADCP; however, randomly oriented particles are already effective at a low dose. This can be explained by a synergy between uptake in macrophages and in cancer cells, leading to an enhanced effect compared to the oriented particles. When directed antibody approaches were used before, uptake was only evaluated in a single cell type, in which an enhanced uptake of the directed particles was observed.^20,47^ This research suggests that using randomly oriented nanoparticles might be beneficial as they are able to trigger the patient’s immune system.

This work expands the toolbox of super-resolution microscopy with probes to characterize antibody domain exposure on NPs, potentially supporting the design of particles with controlled Fab or Fc exposure. Furthermore, the understanding of relationships between structure and targeting capacities in targeted nanomedicine will be improved. Moreover we envision that this DNA-PAINT method can be extended beyond therapeutic NPs to characterize other type of surface-immobilized antibodies, for example in the field of antibody-based biosensors.

## Experimental Section

### Materials

Sicastar COOH (300 nm, 50 mg/mL), Sicastar-greenF COOH (100 nm, 200 nm, 25 mg/mL), Sicastar-redF COOH (200 nm, 25 mg/mL), Sicastar-greenF NH_2_ (200 nm, 25 mg/mL), and Sicastar-redF NH_2_ (200 nm, 25 mg/mL) nanoparticles were obtained from MicroMod. GreenF particles were used in DNA-PAINT experiments, redF particles were used in the ADCP assay. Cetuximab (Erbitux), kanamycin, BugBuster, benzonase were obtained from Merck. *N*-(3-(Dimethylamino)propyl)-*N*′-ethylcarbodiimide hydrochloride (EDC), MES, PBS tablets, chloramphenicol, arabinose, were obtained from Sigma-Aldrich. PD10 columns and HiTrap Q HP columns were purchased at GE Healthcare. QuickChange Lightning multisite-directed mutagenesis kit was obtained from Agilent. *E. coli* BL21(DE3) was purchased at Novagen. β-d-1-Thiogalactopyranoside was obtained from IPTG. Unnatural amino acid p-BpA was obtained from Bachem. Desthiobiotin originates from IBA Life Sciences Zeba spin columns (0.5 mL, 7 kDa MWCO) and sulfosuccinimidyl 4-(*N-*maleimidomethyl) cyclohexane-1-carboxylate (Sulfo-SMCC) were purchased from ThermoFisher Scientific. NH_2_–ODN dockings and I1 were obtained from Integrated DNA Technologies, and IPS3 was obtained from Eurofins. ODNs were dissolved in storage TE buffer and used fresh. The sequences of ODNs used are listed in Table 1. Specific buffer was used for imaging. Buffer B+: 10 mM MgCl_2_, 54 mM Tris-HCl, 1 mM EDTA, 0.05 v/v% Tween 20, pH 8.

**Table 1:** Overview of DNA Strands Used

name	sequence
Docking strand 1 (D1)	3′-ATC TAC ATA TT/5AmMC6/-5′
Imager strand 1 (I1)	5′-CTA GAT GTA T/3ATTO647NN/-3′
Docking strand PS3 (DPS3)	3′-/3AmMO/TTA GGA GGG-5′
Imager strand PS3 (IPS3)	5′-/Cy3B/TCC TCC C-3′

### Optical Setup

DNA-PAINT imaging was carried out with the Oxford Nano Imager (ONI). Focus was maintained using the focus laser of the Nanoimager. By irradiation under total internal reflection (TIR) conditions, fluorescence emission could be observed selectively from particles on the glass slide, and thus with a reduced background. After laser excitation (542 and 640 nm), the fluorescent signal was collected by a 100× 1.4 NA oil immersion objective (Olympus), passed through a dichroic mirror and was collected by an ORCA Flash 4 sCMOS (Hamamatsu) camera. A movie was obtained, where the blinking by molecule emission is visualized as circular spots lasting a few acquisition frames.

### Recombinant Protein Cloning, Expression, and Purification

Both protein G (pG) and protein M (pM) were encoded in pET28a vectors which were synthesized by GenScript. The pG construct was based on a synthetic monomeric variant containing a C-terminal cysteine and a photoactive unnatural amino acid for covalent cross-linking to antibodies (Figure S16).^48^ The pM construct was based on the truncated TD variant of protein M described by Grover et al.^41^ A single cysteine was incorporated by site-specific mutation of N235, located in a flexible internal loop pointing away from the antigen binding site (Figure S17). For this, the QuikChange Lightning Multi Site-Directed Mutagenesis kit (Agilent) was used according to manufacturer’s instructions, using primer 5′-GGCTCACCTCTGTATGATAGCTACCCTTGTCATTTTTTTGAAGATGT-3′.

#### Protein Expression of pG

The expression plasmid was cotransformed with the pEVOL-pBpF plasmid (kind gift from Peter Schultz, Addgene plasmid no. 31190), which contains a tRNA–tRNA synthetase pair enabling incorporation of *p*-benzoylphenylalanine (pBzF), in chemically competent *Escherichia coli* BL21(DE3) cells (Novagen). Bacteria were cultured in 0.5 L 2xYT medium (2.5 g of NaCl, 5 g of Yeast extract, 8 g of Peptone in 0.5 L dH2O) supplemented with 50 μg/mL kanamycin and 25 μg/mL chloramphenicol. When the OD_600_ reached 0.5–0.6, expression was induced using 1 mM isopropyl β-d-1-thiogalactopyramoside (IPTG), 0.02 w/v % arabinose and 1 mM pBzF. After overnight expression at 20 °C and 250 rpm, cells were harvested by centrifugation at 10,000*g* for 10 min. Cells were then lysed using BugBuster protein extraction reagent (Novagen) and Benzonase endonuclease (Novagen) for 1 h and subsequently centrifuged at 16,000*g* for 20–40 min.

#### Protein Expression of pM

The expression plasmid was transformed in chemically competent *Escherichia coli* BL21(DE3) cells and cultured in 0.5 L 2xYT medium (2.5 g of NaCl, 5 g of Yeast extract, 8 g of Peptone in 0.5 L dH_2_O) supplemented with 50 μg/mL kanamycin. When the OD_600_ reached 0.5–0.6, expression was induced using 1 mM IPTG. After overnight expression at 20 °C and 250 rpm, cells were harvested by centrifugation at 10,000*g* for 10 min. Cells were then lysed using BugBuster protein extraction reagent (Novagen) and Benzonase endonuclease (Novagen) for 1 h and subsequently centrifuged at 16,000*g* for 20–40 min.

Both proteins were purified using subsequent Ni-affinity chromatography (Novagen, His-bind resin) and Strep-Tactin XT (IBA) purification according to the manufacturer’s instructions. To this end, the cleared lysate was applied to a nickel-charged column, washed with wash buffer (1× PBS, 370 mM NaCl, 10% (v/v) glycerol, 20 mM imidazole, pH 7.4), and eluted with elution buffer (1× PBS, 370 mM NaCl, 10% (v/v) glycerol, 250 mM imidazole, pH 7.4). The eluate was then applied to a Strep-Tactin column. The column was washed with wash buffer (100 mM Tris-HCl, 150 mM NaCl, 1 mM EDTA, pH 8.0) and the protein eluted with wash buffer supplemented with 50 mM biotin. Proteins were aliquoted in 500-μL fractions and stored frozen at −80 °C until further use. Absorption at 280 nm (ND-1000, Thermo Scientific) was used to calculate protein concentration, assuming extinction coefficients of 58,220 and 15,570 M^–1^ cm^–1^ for pM and pG, respectively. Purity was assessed on 4–20% SDS-PAGE precast gels (Bio-Rad) under reducing conditions, stained with Coomassie Brilliant Blue G-250 (Bio-Rad) (Figure S18A,B). The molecular weight was confirmed using liquid chromatography quadrupole time-of-flight mass spectrometry (Waters ACQUITY UPLC I-Class System coupled to a Xevo G2 Q-ToF) by injecting a 0.1 μL sample into an Agilent Polaris C18A reversed-phase column with a flow of 0.3 mL min^–1^ and a 15–60% acetonitrile gradient containing 0.1% formic acid (Figure S18C,D).

### Preparation of Reaction ODNs

To 100 μL of amine-functionalized ODN (200 μM in 1× PBS, pH 7.4) was added 100 μL sulfo-SMCC (2 mM in dry DMSO) to obtain a final ODN concentration of 100 μM. The mixture was incubated at 25 °C at 850 rpm for 2 h. Two rounds of ethanol precipitation were used to remove excess sulfo-SMCC. The SMCC-labeled ODNs were precipitated by adding 10% (v/v) 3.0 M potassium acetate, pH 5.5 (Buffer P3, Qiagen) and 300% (v/v) ice-cold EtOH and incubation for 60 min at −30 °C. After centrifugation at 19,000*g* for 30 min at 4 °C, the pellet was resuspended in 1× PBS (pH 7.4), and the precipitation and centrifugation were repeated. The pellet was washed in 95% (v/v, in water) ice-cold EtOH, centrifuged at 19,000*g* for 15 min, and dissolved in TE buffer (10 mM Tris, 1 mM EDTA, pH 8.0). Absorption at 260 nm (ND-1000, Thermo Scientific) was used to calculate DNA concentration, assuming extinction coefficients of 110,600 and 94,300 M^–1^ cm^–1^ for D1 and DPS3, respectively. DNA was stored at −30 °C until further use.

### Conjugation of ODN to Protein (pG/pM)

Before conjugation, aliquots of pG and pM were thawed and the proteins were reduced by adding TCEP to a final concentration of 5 mM and incubating at 25 °C for 1 h at 400 rpm. The protein solutions were buffer-exchanged to reaction buffer (100 mM sodium phosphate supplemented with 25 μM TCEP, pH 7) using a PD10 desalting column. Immediately after, conjugation reactions were carried out on a 600–800 μL scale using 20 μM protein and 60 μM SMCC–ODN in reaction buffer for 2 h at 25 °C at 400 rpm. To remove excess SMCC–ODN, Strep-Tactin affinity chromatography was performed as described above. To remove unreacted protein, small-scale ion-exchange chromatography was performed using 0.5 mL strong anion-exchange spin columns (Thermo Scientific). After equilibration with purification buffer (100 mM Tris-HCl, 150 mM NaCl, 1 mM EDTA, pH 8.0), the protein mixture was directly loaded onto the column in 400 μL fractions, according to the manufacturer’s instructions. Elution was performed by stepwise increase of the NaCl concentration in the purification buffer (at 200, 300, 400, 500, 600 mM, in turn). Typically, the protein eluted at <300 mM NaCl, whereas enzyme–ODN conjugates eluted at 500–600 mM NaCl. Elution fractions containing pure enzyme–ODN conjugates were pooled, snap-frozen in liquid nitrogen and stored at −80 °C in 5 μL aliquots.

### Antibody Functionalization of Carboxylic Acid Nanoparticles

Conjugation of cetuximab to NPs was performed as reported previously.^18^ From the commercial suspension of carboxylic acid NPs, 0.06 mg 300 nm particles were washed with 500 μL 50 mM MES buffer pH = 5.0 by centrifugation at 16,900*g* for 15 min. For activation, the particles were resuspended in MES buffer containing 39 nmol EDC, in a total volume of 60 μL, with a particle concentration of 1 mg/mL. The solution was kept under moderate shaking for 15 min at room temperature. Ctx was buffer exchanged to 1× PBS using Zeba spin columns following the manufacturers protocol. Briefly, the storage buffer was removed (1,500*g*, 1 min), after which 1× PBS was added to the resin bed and the column was washed 3× (1,500*g*, 1 min). The column was placed in a collection tube and Ctx was added directly to the top of the resin bed. Buffer exchanged Ctx was obtained by centrifuging 2 min, 1,500*g* and the final concentration was calculated on the basis of absorption at 280 nm, assuming an extinction coefficient of 210,000 M^–1^ cm^–1^. Then, for the antibody coating, the particles were further diluted twice with MES buffer and 0.104 nmol Ctx (0.68 Ctx/COOH) was added in a total volume of 125 μL. This solution was kept under moderate shaking for 2 h at room temperature before washing three times with 1× PBS for the removal of unconjugated antibodies (16,900*g*, 15 min). Finally, the pellet of Ctx-labeled NPs was reconstituted in 1× PBS to reach a final concentration of 1 mg/mL and stored in the dark at 4 °C.

In the case of 100 nm carboxylic acid NPs, 0.09 mg particles was used. Furthermore, both for 100 and 200 nm beads, 0.073 nmol Ctx was added.

### pG or pM Functionalization of Amino Nanoparticles

For conjugation of either pM or pG to the surface of amino NPs, the proteins were first reduced using TCEP. To 1 mL of protein, 5 mM TCEP was added and this solution was incubated for 1h at 25 °C, under moderate shaking. Thereafter, the protein was desalted using PD-10 columns and reaction buffer (100 mM sodium phosphate, 25 μM TCEP, pH 7) and the concentration was determined on the basis of absorption at 280 nm, assuming an extinction coefficient of 58,220 M^–1^ cm^–1^ and 15,470 M^–1^ cm^1^ for pM and pG respectively. Meanwhile, a solution of 1 mg 200 nm green fluorescent amino NPs (40 μL) was concentrated to a concentration of 100 mg/mL in 1× PBS, pH 7.2 (10 μL) and 10 nmol Sulfo-SMCC in DMSO (10 μL) was added. The reaction was incubated for 30 min at room temperature with slow rotation and tilt. Excess Sulfo-SMCC was removed by washing with 400 μL reaction buffer (16,500*g*, 5 min). The NPs were resuspended in 100 μL reaction buffer containing a total protein concentration of 100 μM, with either a 0:1, 1:3, 1:1, 3:1 or 1:0 pG:pM molar ratio. After 2 h of incubation, the NPs were washed with 1× PBS (16,500*g*, 5 min, 2×). Finally, the pellet of protein-labeled NPs was reconstituted in 1× PBS to reach a final concentration of 25 mg/mL and stored in the dark at 4 °C.

### Antibody Functionalization of pG or pM Nanoparticles

For oriented conjugation of Ctx to the surface of pG or pM labeled NPs, the NPs were sonicated to obtain a homogeneous suspension of the particles. The concentration was further diluted to a 2 mg/mL particle solution in 1× PBS. Then, 0.1 nmol Ctx was added to the solution and this suspension was incubated for 2 h with moderate shaking, protected from the light. Excess Ctx was removed by centrifuging once (16,900*g*, 15 min). The pellet was resuspended in 1× PBS to reach a final concentration of 1 mg/mL.

### pG-ODN and pM-ODN Labeling

Labeling of the Fc and Fab domains was achieved by incubating the NPs with pG-ODN and pM-ODN, either together or separately. First, the labeled NPs were sonicated to ensure resuspension of the particles. To 15 μg of the antibody labeled NPs, 24.8 pmol pG and 12.5 pmol pM (approximately 1:4 and 1:8 compared to amount of Ctx) were added and this was incubated for 1 h under moderate shaking at room temperature. For the removal of excess probes, the particles were washed once using 1× PBS (16,900*g*, 15 min). The pellet was resuspended in 1× PBS to reach a final concentration of 0.38 mg/mL. To prevent aggregation, the particles were sonicated for 5 min.

### NP Quantitative Imaging Acquisition

For acquisition, an imager mix was prepared, containing 1.6 nM I1 and 0.8 nM IPS3 in Buffer B+. The NPs were loaded into a Ibidi μ-Slide VI 0.5 Glass Bottom flow chamber and they were allowed to adsorb for 10 min. For the oriented particles (pG and pM covered), the Ibidi slides were cleaned with UVOzone (Novascan) for 15 min prior to loading of the NPs. Particles that were not adsorbed on the glass were washed away using 1× PBS. Subsequently, the imager mix was flowed into the chamber and the chamber was closed with caps to prevent evaporation. TIR conditions were used for all qPAINT experiments. For the green fluorescent particles, one frame with the location of the particles was collected in the 473 channel (exposure time 90 ms, power ∼1 mW). To avoid background signal from this fluorescence, the NPs were bleached for 10 min at full laser power (∼235 mW). Following this, DNA-PAINT was performed using the 532 and 640 laser (power 65 mW and 40 mW respectively) simultaneously for the excitation of the imager strands. Emission was detected in multiple 428 × 500 μm ROIs for 10,000 frames at a rate of 11 Hz, with a total acquisition time of 15 min.

### Kinetics Calibration

qPAINT uses kinetic information derived from the mean dark time (τ_d*_) between binding events and by using *n* = (*k*_on_*c*_i_τ_d*_)^−1^, the number of ligands is obtained. The second-order association rate between the docking and imager strand (*k*_on_) needs to be calibrated. To achieve this, PLL-biotin was immobilized on the glass surface for 5 min, after which streptavidin was added and incubated for 5 min. Biotin-docking was added and incubated for 10 min. Excess was washed away with 1× PBS in between steps. By adding a known imager concentration (*c*_i_) of 2 nM in Buffer B+, the *k*_on_ of a single docking strand and imager strand pair can be calculated. These values were calculated to be 3.0 × 10^6^ M^–1^ s^–1^ and 4.1 × 10^6^ M^–1^ s^–1^ for D1-I1 and DPS3-IPS3 respectively. These values are similar to previously reported values.^29−31,49^

### Analysis

The Nanoimager software generates a list of events of binding dyes in the acquired movie. These lists were analyzed using Matlab R2021a script. To start with, a Matlab script was used to make the obtained csvs ThunderSTORM compatible, thereafter, the files were loaded into ThunderSTORM v1.3^50^ and density filtered (100 neighbors in 100 nm for 300 nm, 50 neighbors in 100 nm for 200 nm and 40 neighbors in 100 nm for 100 nm particles). These files were exported and by using Matlab, the locations were merged into one color, saving the channel name for each, after which a mean-shift clustering algorithm was applied in order to identify clusters of localizations that are NPs. An additional filter was applied to make sure there were no aggregates nor clusters with unrealistic shape in the eventual data. The mass center of the resulting clusters was calculated. Then, the localizations were split according to their channel number and the (*x*,*y*,*t*) coordinates were stored for each NP and each channel. With these coordinates, a binary intensity versus time trace was reconstructed for each NP and channel, thus assigning a value of 0 to frames without localizations and 1 to frames with one localization. The 0 values correspond to dark times, and these were separated for each NP, which resulted in the empirical cumulative distribution function (CDF), which was then fitted to the exponential model following *y*(*t*) = 1 – *Ae*^–*t*/τd*^. By doing this, the value of the mean dark time τ_d*_ was obtained, and this value was used to calculate the number of Fc and Fab for each NP using *n* = (*k*_on_*c*_i_τ_d*_)^−1^.

### Cell Viability Assay

A431 cells were harvested and seeded in a 96 well plate at a density of 30,000 cells well together with the different sicastar-redF functionalized NPs at a concentration of 150 μg/mL and a 10% of Alamar Blue HS. After 4 h of incubation at 37 °C and 5% CO_2_, the fluorescence emission (590 nm) was measured in a Varioskan (Thermo Scientific) at 560 nm excitation. Mean fluorescence intensity and standard deviation of 4 replicates is plotted.

### ADCP Assay

Human CD14^+^ monocytes were isolated from buffycoats of healthy donors (Sanquin blood Supply, Amsterdam, The Netherlands) with magnetic cell separation beads (Miltenyl Biotech) according to the manufacturer’s protocol. By culturing the isolated CD14^+^ blood monocytes with 50 ng/mL macrophage-colony stimulating factor (M-CSF) (eBioscience) for 6 days in RPMI 1640 (Gibco) supplemented with 10% heat inactivated fetal calf serum (FCS, Lonza), glutamine (Lonza) and penicillin/streptomycin (Lonza), macrophages were obtained. These cells were stained with DiO (Molecular Probes Inc.) according to the manufacturer’s protocol and seeded in a 24 well plate at 150,000 cells per well (E:T ratio 5:1). A431 cells were labeled with eFluor 670 (eBioscience). These were seeded on top of the macrophages (30,000 cells per well) together with the different, sicastar-redF functionalized NPs at a concentration ranging between 10 and 150 μg/mL. As a positive control for phagocytosis, cetuximab (10 ng/mL) was used. As a negative control, sicastar-redF 200 nm COOH and NH_2_ were added. After 4h of incubation at 37 °C, 5% CO_2_, the supernatant was collected in falcon tubes and cells were washed with PBS (+ Ca^2+^ + Mg^2+^). Macrophages and tumor cells were detached with 250 μL of trypsin and a cell scraper and added to the falcon tubes. The cell suspension was washed twice with PBS supplemented with calcium, magnesium and 0.2% BSA and analyzed by flow cytometry. During analysis, the debris was excluded from the sample, after which the signal in the blue channel (DiO) was plotted over the red channel (eFluor 640) and gated for double positive cells. This double positive quadrant was used for further processing by plotting its signal in the yellow (redF) channel for the triple positive cells. Percentage of triple positive cells was calculated by dividing the number of triple positive cells over the total number of cells.

## Supporting Information Available

Experimental methods; protein characterization data; bare particle controls; larger fields of view DNA-PAINT; domain exposure dependency on size; number of domains measured by DNA-PAINT; geometrical model of antibody accessibility; viability assay of A431 cells; double positive cells per conjugation strategy; ADCP for bare particles (PDF)
