# Supplementary material for: Mapping Antibody Domain Exposure on Nanoparticle Surfaces Using DNA-PAINT
Source: ACS Nano. 2023 Jun 7;17(12):11665–78. doi: 10.1021/acsnano.3c02195 (PMC10311592; doi:10.1021/acsnano.3c02195)
Supplement: Supplementary file 1 — nn3c02195_si_001.pdf [file nn3c02195_si_001.pdf]

Supporting information

Mapping Antibody Domain Exposure on Nanoparticle Surfaces Using DNA-PAINT

Marrit M.E. Tholen[a], Bas J.H.M. Rosier[a], Robin T. Vermathen[a], Céline A.N. Sewnath[b,c,d], Cornelis Storm[e], Laura Woythe[a], Cristina Izquierdo-Lozano[a], Roger Riera[a], Marjolein van Egmond[b,c,d,f], Maarten Merkx[a], Lorenzo Albertazzi[a]*

[a] Department of Biomedical Engineering, Institute for Complex Molecular Systems (ICMS), Eindhoven University of Technology, 5612 AZ Eindhoven, The Netherlands.

[b] Department of Molecular Cell Biology and Immunology, Amsterdam UMC, Vrije Universiteit Amsterdam, De Boelelaan 1117, 1081 HV, Amsterdam, The Netherlands.

[c] Cancer Center Amsterdam, Cancer Biology and Immunology De Boelelaan 1117, 1081 HV, Amsterdam, The Netherlands.

[d] Amsterdam institute for Infection and Immunity, Cancer Immunology, De Boelelaan 1117, 1081 HV, Amsterdam, The Netherlands.

[e] Department of Applied Physics, Institute for Complex Molecular Systems (ICMS), Eindhoven University of Technology, 5612 AZ Eindhoven, The Netherlands.

[f] Department of Surgery, Amsterdam UMC, Vrije Universtiteit Amsterdam, De Boelelaan 1117, 1081 HV, Amsterdam, The Netherlands.

Supplementary Figures

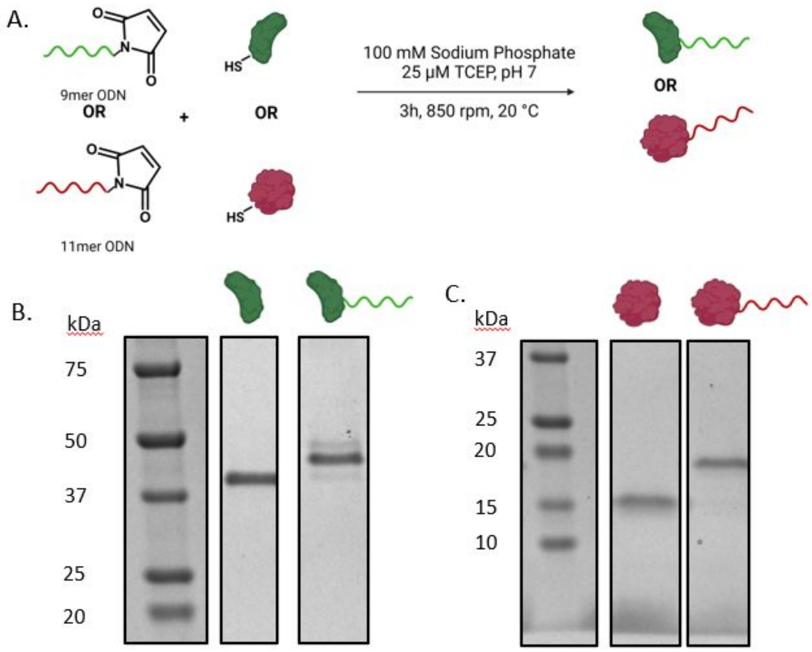

Figure S 1: (A) Schematic overview and reaction conditions for pM-ODN and pG-ODN coupling. (B) SDS-PAGE gel analysis under non-reducing conditions of the pM without ODN (center) and the pM-ODN conjugate after purification (right, see Methods). Addition of a 5-fold molar excess of 3'maleimide-functionalized 9-mer ODN and purification using small-scale ion-exchange chromatography resulted in >95% pure conjugate product. (C) SDS-PAGE gel analysis under non-reducing conditions of pG without ODN (center) and the pG-ODN conjugate after purification (right, see Methods). Addition of a 5-fold molar excess of the 5'-maleimide-functionalized 11-mer ODN and purification using small-scale ion-exchange chromatography resulted in >95% of pG labelled with ODN. Schematic was created with BioRender.com

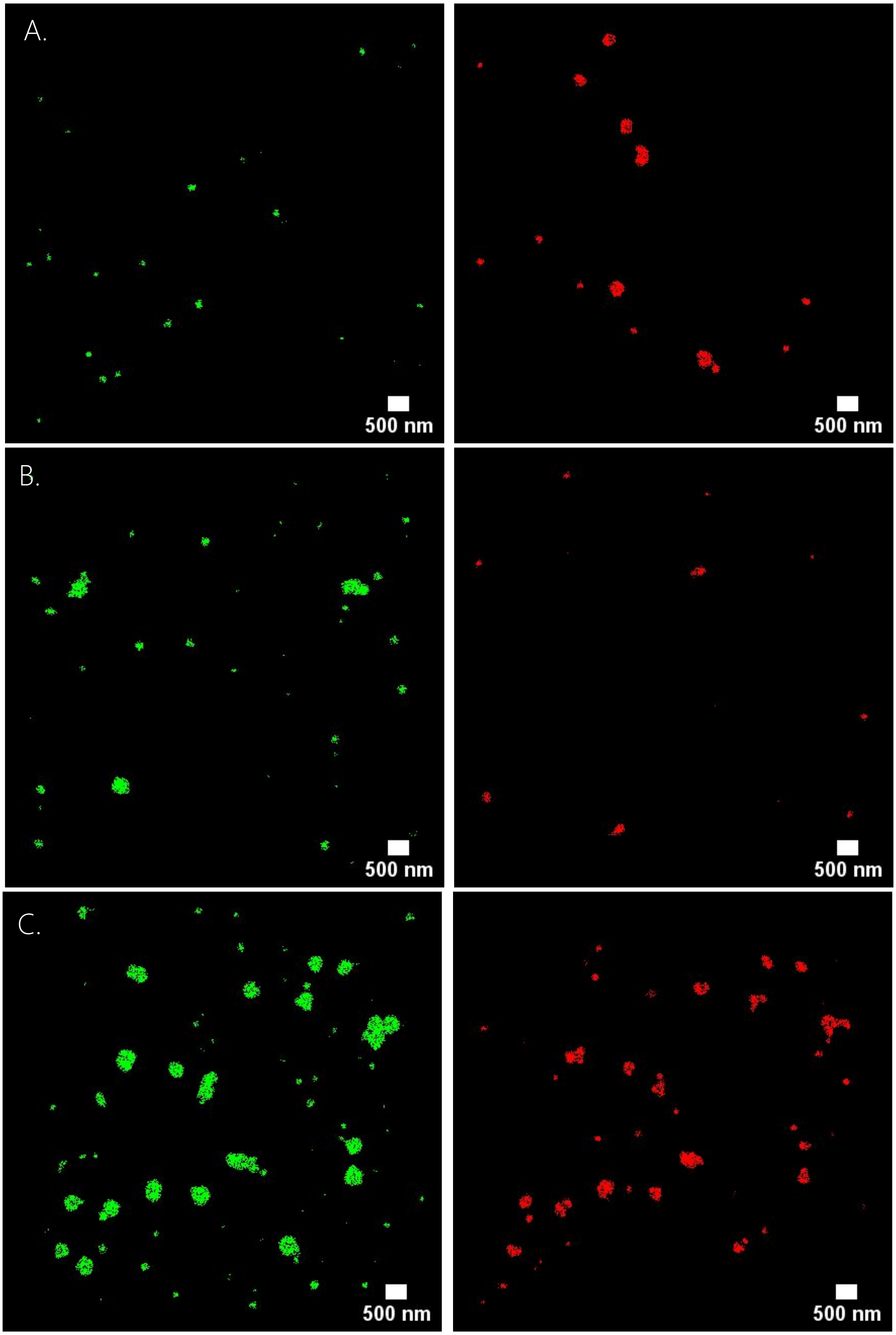

Figure S 2: Separate channels of the images found in Figure 2. Left: pM channel (0.8 nM IPS3), right: pG channel (1.6 nM I1). (A) 300 nm random Ctx particles incubated with 24.8 pmol pG probes. (B) 300 nm random Ctx particles incubated with 12.5 pmol pM probes. (C) 300 nm random Ctx particles incubated with both pG and pM. Scale bars: 500 nm

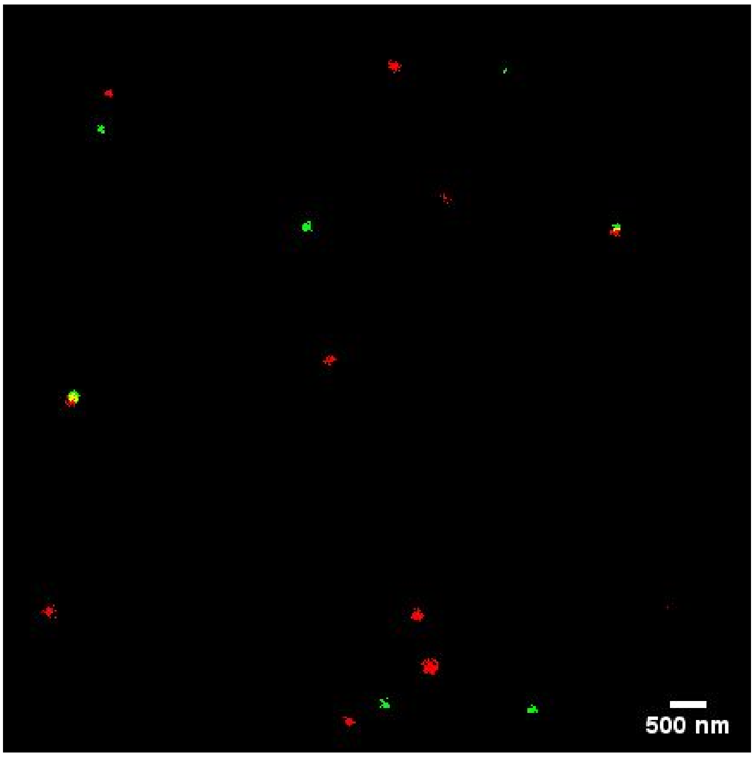

Figure S 3: Blank measurement of Sicastar 300 nm COOH particles with both imagers present (1.6 nM I1 and 0.8 nM IPS3), but no antibodies. Scale bar: 500 nm

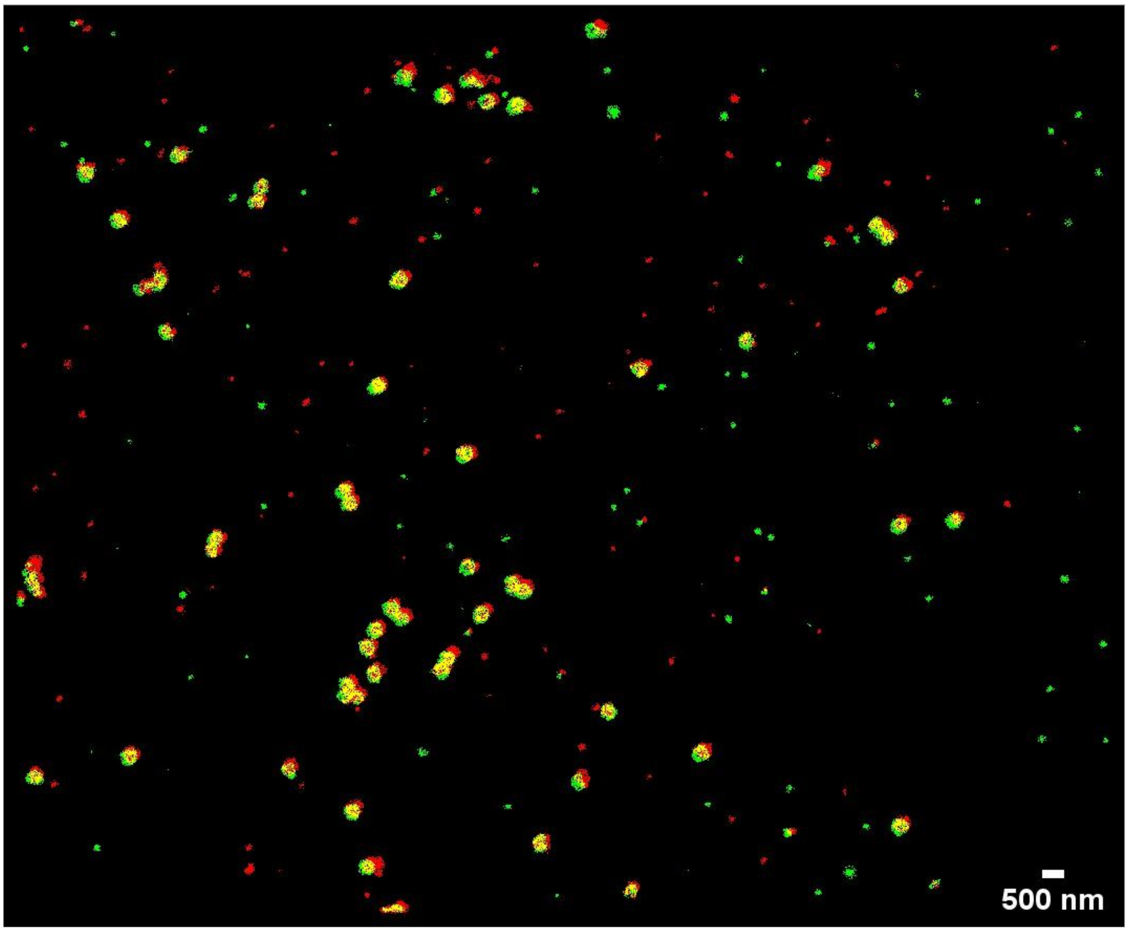

Figure S 4: Larger field of view of 300 nm random Ctx particles incubated with both pG and pM probes (24.8 pmol and 12.5 pmol respectively). Imaged with an imager mix containing 1.6 nM I1 and 0.8 nM IPS3 Scale bar: 500 nm.

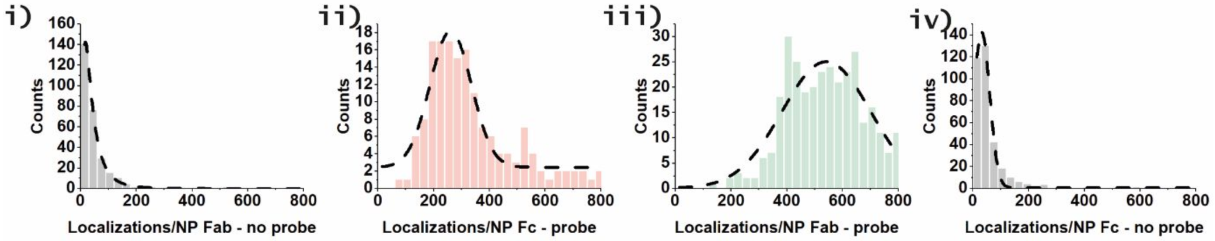

Figure S 5: Number of localizations per random nanoparticle, measured with DNA-PAINT, but only one of the probes mixed in and processed using a clustering algorithm (Figure 2). i) number of localizations in the green channel with only pG present, ii) number of localizations in the red channel, with only pG present, iii) number of localizations in the green channel with only pM present, iv) number of localizations in the red channel with only pM present. Distributions were fitted with a Gaussian distribution to extract the mean.

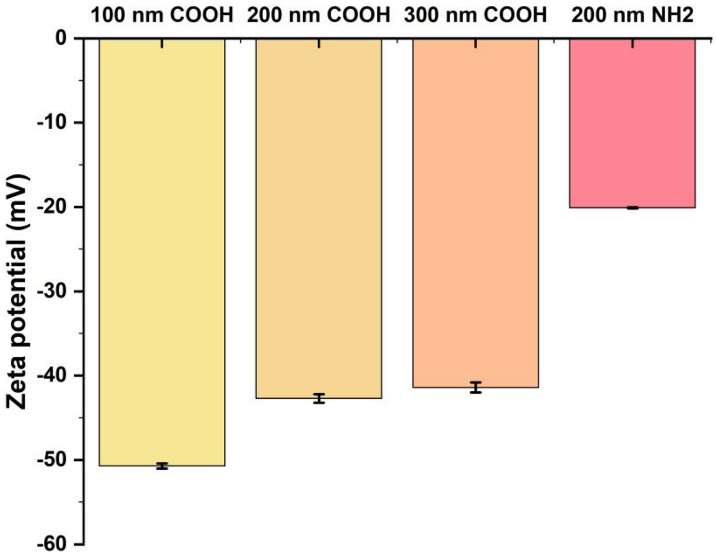

Figure S 6: Zeta potential of Sicastar particles without functionalization.

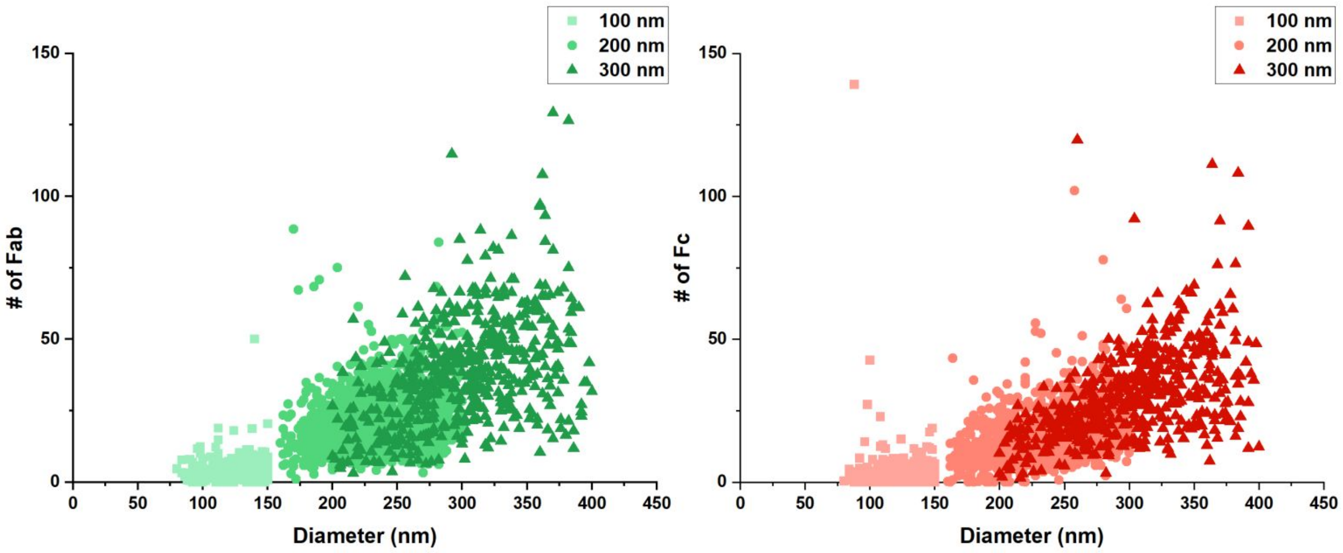

Figure S 7: Number of domains on the random Ctx particles plotted over size. A) Number of Fab domains, obtained from the clustering algorithm, plotted over reported sizes. B) Number of Fab, obtained from the clustering algorithm, plotted over reported sizes.

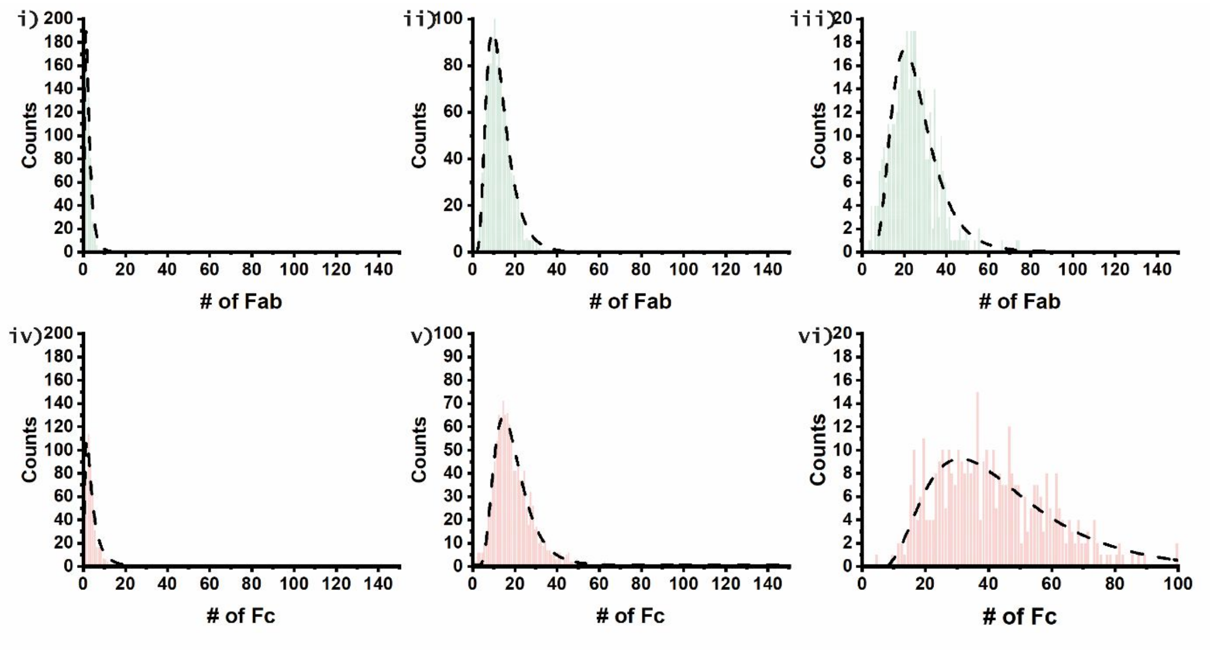

Figure S 8: Number of domains per random nanoparticle, measured with DNA-PAINT and processed using a clustering algorithm (Figure 3). i) number of Fab 100 nm, ii) number of Fab 200 nm, iii) number of Fab 300 nm, iv) number of Fc 100 nm, v) number of Fc 200 nm, vi) number of Fc 300 nm. Distributions were fitted with a Log normal distribution to extract the mean.

Table S 1: Coefficient of Variation (%, CV) of the number of domains per NP measured by DNA-PAINT for the random NPs (Figure 3). Histograms in Figure S6 were fitted with a Log normal distribution to extract the mean, standard deviation and R-square.

Sample	Domain	Number of particles	Fitting	R-square	Mean	SD	% Coefficient of Variation (CV)
100 nm	Fab	527	Log Normal	0.99	2.7	2.2	81.5
	Fc	527	Log Normal	0.94	5.0	5.40	108
	Size	527	Log Normal	0.35	138.0	11.8	8.6
	Surface area	527	Gaussian	0.89	54985	16224	29.5
200 nm	Fab	1195	Log Normal	0.99	13.3	6.8	51.1
	Fc	1195	Log Normal	0.97	19.4	8.9	45.9
	Size	1195	Log Normal	0.39	236	37.2	15.8
	Surface area	1195	Gaussian	0.91	166965	51503	30.8
300 nm	Fab	409	Log Normal	0.91	26.4	11.7	44.3
	Fc	409	Log Normal	0.81	47.7	27.4	57.4

	Size	409	Log Normal	0.36	317	41.1	13.0
	Surface area	409	Gaussian	0.95	288100	92235	32.0

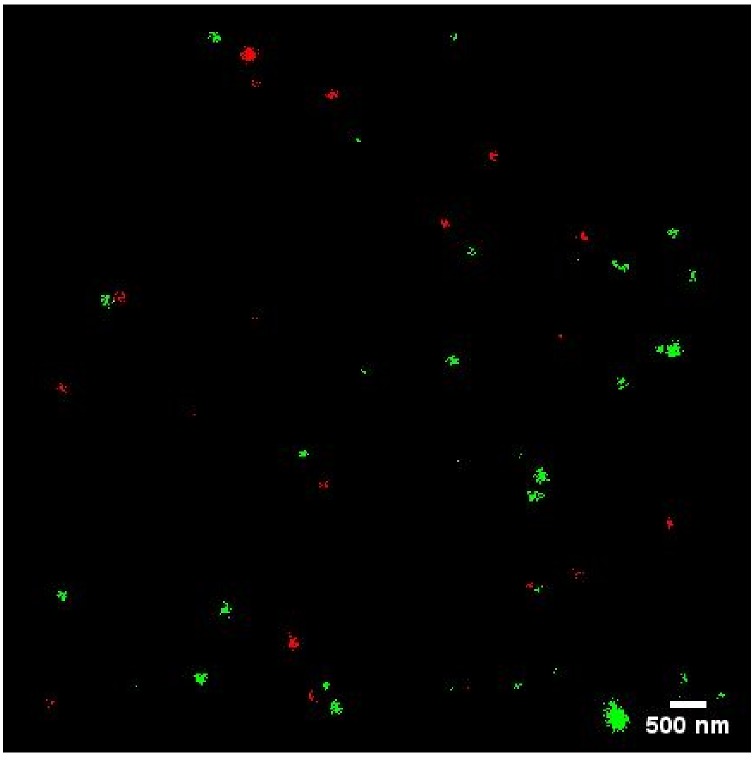

Figure S 9: Blank measurement of Sicastar greenF 200 nm NH_2 particles with both imagers (1.6 nM I1 and 0.8 nM IPS3), but no antibody present. Scale bar: 500 nm.

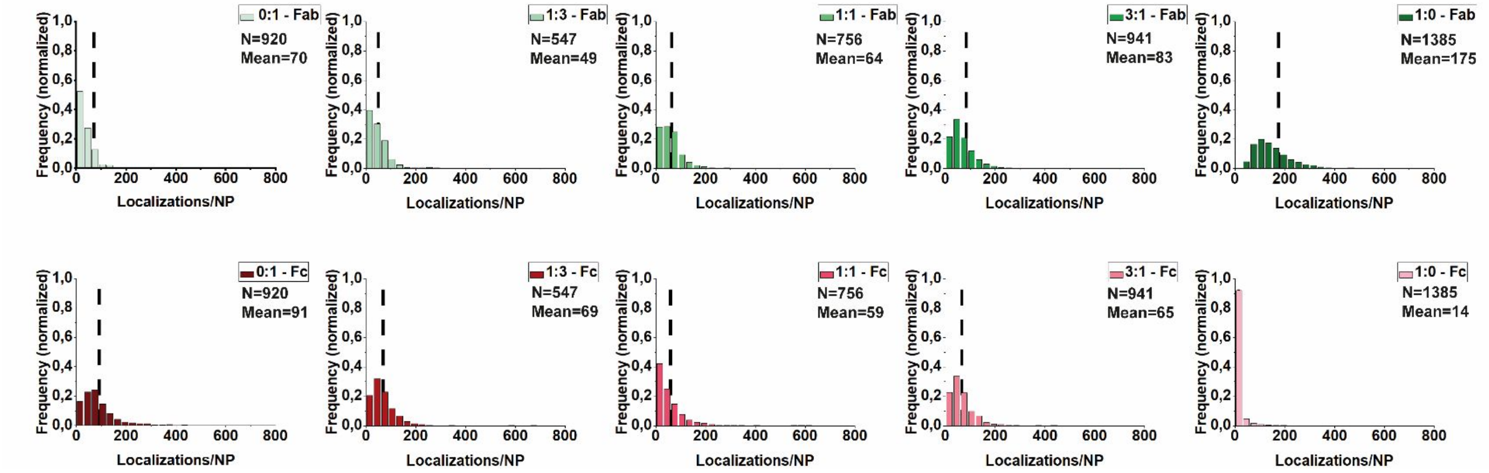

Figure S 10: Overview of the localizations/NP obtained from the ratio particles. Incubated with 24.8 pmol pG and 12.5 pmol pM and imaged with an imager mix containing 1.6 nM I1 and 0.8 nM IPS3. Number of localizations obtained using a clustering algorithm. Top: number of localizations for Fab domains, bottom: number of localizations for Fc domains.

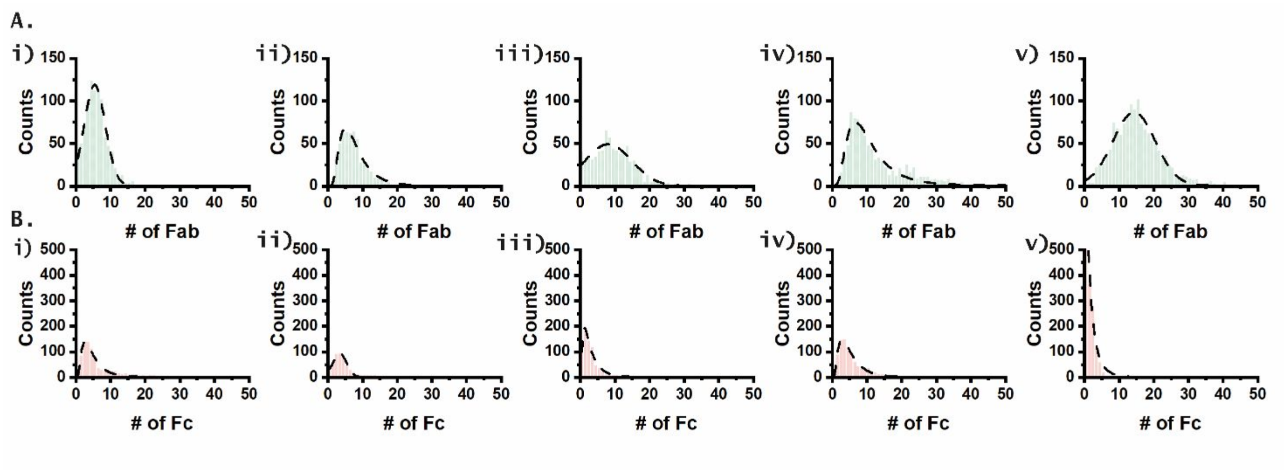

Figure S 11: Number of domains per nanoparticle, measured with DNA-PAINT. i) number of Fab 100 nm, ii) number of Fab 200 nm, iii) number of Fab 300 nm, iv) number of Fc 100 nm, v) number of Fc 200 nm, vi) number of Fc 300 nm. Distributions were fitted with a Log normal distribution or a Gaussian (Table S2) to extract the mean.

Table S 2: Coefficient of Variation (%, CV) of the number of domains per NP measured by DNA-PAINT. Histograms in Figure S11 were fitted with a Log normal distribution to extract the mean, standard deviation and R-square.

**Sample**	**Domain**	**Number of particles**	**Fitting**	**R-square**	**Mean**	**SD**	**% Coefficient of Variation (CV)**
0:1	Fab	920	Gaussian	0.99	7.2	3.1	43.1
	Fc	920	Log Normal	0.97	5	3.6	72
1:3	Fab	547	Log Normal	0.98	8	4.7	58.8
	Fc	547	Gaussian	0.97	4.6	2.0	43.0
1:1	Fab	756	Gaussian	0.96	15.8	6.7	52.4
	Fc	756	Log Normal	0.99	3.6	3.6	100
3:1	Fab	941	Log Normal	0.96	11	6.9	62.7
	Fc	941	Log Normal	0.98	5.7	4.3	75.4
1:0	Fab	1385	Gaussian	0.97	14.4	6.1	42.4
	Fc	1385z	Log Normal	0.98	2.5	2.9	116

Computational model details.

Orientations and parameter values.

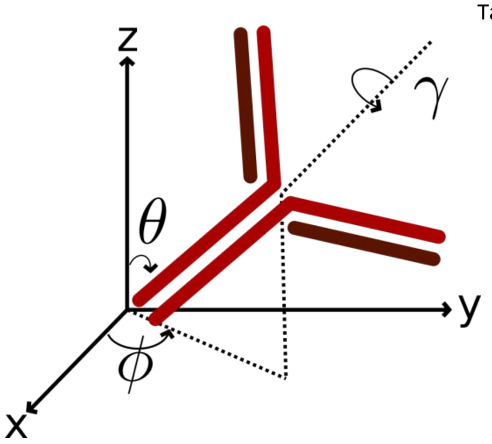

Table S 3: Clarification of parameters used in the computational model

Parameter	Description	Value
R	NP radius	40-160 nm
l_{fc}	Fc stalk length	8 nm
l_{fab}	Fab stalk length	6.5 nm
θ_{fab}	Fab opening angle	100°
$r_{M,excl}$	pM exclusion zone radius	4 nm
$r_{G,excl}$	pG exclusion zone radius	4 nm
θ	Azimuthal angle	$0-\pi$ rad
ϕ	Polar angle	$0-2\pi$ rad
γ	Axial rotation angle	$0-\pi$ rad

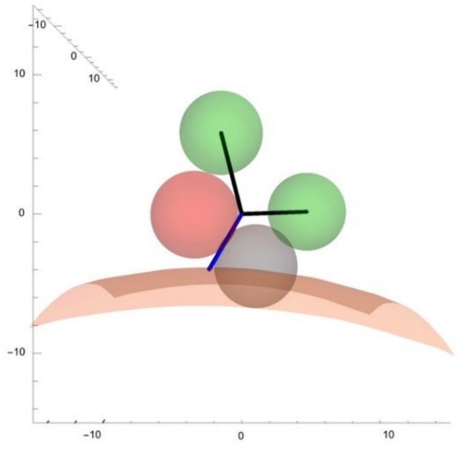

Figure S 12: One binding orientation showing available and occluded protein binding domains. Angle values: θ=.68, ϕ=.77, γ=1.33. Particle radius = 50nm. For this configuration, both pM binding domains are available (nM=2, green blobs), and one of the pG binding domains is occluded (grey blob) by the particle. The other pG binding domain is available (nG=1, red blob).

Statistics of random conjugation.

The antibody orientation is parameterized by three Euler angles. Each combination of angles is a unique binding orientation, and we compute which of the three end points has the lowest z-coordinate and use this as the nanoparticle-antibody conjugation site. There are thus three possible conjugation sites, two of which are Fab-epitopes and one is the Fc stalk end. Integrating over all possible orientations allows us to compute the phase space volumes corresponding to Fab-bound and Fc-bound orientations,

$$V_{Fab} = \frac{1}{4\pi^2}\int_0^{\pi} d\theta \int_0^{2\pi} d\phi \int_0^{\pi} d\gamma \sin\theta \delta_{Fab}(\theta,\phi,\gamma)\ p(\theta,\phi,\gamma),$$

$$V_{Fc} = \frac{1}{4\pi^2}\int_0^{\pi} d\theta \int_0^{2\pi} d\phi \int_0^{\pi} d\gamma \sin\theta \delta_{Fc}(\theta,\phi,\gamma)\ p(\theta,\phi,\gamma),$$

where $\delta_{Fab}(\theta,\phi,\gamma)$ is a function that is equal to one when a particular orientation (θ, ϕ, γ) corresponds to a Fab-bound conjugation, and zero when it corresponds to a Fc-bound conjugation. $\delta_{Fc}(\theta,\phi,\gamma) = 1 - \delta_{Fab}(\theta,\phi,\gamma)$ does the same for Fc-bound states. We assume for now that there is no preference in the orientation of the antibody on NP surfaces, i.e that $p(\theta,\phi,\gamma) = 1$. With this, we can compute the relative probabilities of Fc- and Fab-binding for random conjugation:

$$P_{Fab}^0 = \frac{V_{Fab}}{V_{Fab} + V_{Fc}} = 0.65;\ P_{Fc}^0 = 1 - P_{Fab}^0 = 0.35.$$

Thus, for truly random conjugation 35% will be conjugated at the Fc end, and 65% will be conjugated at the Fab end. These numbers do not depend on NP radius.

Statistics of binding site accessibility.

For any given binding orientation, the number of accessible protein G binding sites, n_G is given by $n_G = a_{G,1} + a_{G,2}$ where $a_{G,i}$ is the accessibility of protein G binding site $i = 1,2$ given by

$$a_i = \begin{cases} 1 & \text{if } d_{G,i}(\theta,\phi,\gamma)\ \geq r_{G,excl} + R \\ 0 & \text{otherwise.} \end{cases}$$

Above, $d_{G,i}(\theta,\phi,\gamma)$ is the distance between the center of protein G exclusion zone i, and the center of the NP. The NP is placed such that it touches the end of the antibody with the smallest z-coordinate value in the axes shown in Figure S12. The number of available protein M binding sites for a given orientation is computed in completely analogous fashion.

To compute the average availability of pM/pG binding sites, we make the simplifying assumption that binding sites can only be occluded by the NP, not by neighboring Abs. This assumption is validated by the low observed areal coverage which is only about 4.2%. We are thus allowed to assume that overlap between antibodies is negligible, which reduces the task of computing the averaged numbers of accessible protein binding sites $\langle n_G \rangle$ and $\langle n_M \rangle$ over all antibodies to computing the same average over all the orientations θ, ϕ, and γ of a *single* antibody. Splitting these averages out into Fab- and Fc-bound configurations, we compute the four key averages

$$\langle n_G \rangle_{Fab} = \frac{1}{4\pi^2}\int_0^{\pi} d\theta \int_0^{2\pi} d\phi \int_0^{\pi} d\gamma \sin\theta \delta_{Fab}(\theta,\phi,\gamma)\ n_G(\theta,\phi,\gamma)\ p(\theta,\phi,\gamma).$$

$$\langle n_G \rangle_{Fc} = \frac{1}{4\pi^2}\int_0^{\pi} d\theta \int_0^{2\pi} d\phi \int_0^{\pi} d\gamma \sin\theta \delta_{Fc}(\theta,\phi,\gamma)\ n_G(\theta,\phi,\gamma)\ p(\theta,\phi,\gamma).$$

$$\langle n_M \rangle_{Fab} = \frac{1}{4\pi^2}\int_0^{\pi} d\theta \int_0^{2\pi} d\phi \int_0^{\pi} d\gamma \sin\theta \delta_{Fab}(\theta,\phi,\gamma)\ n_M(\theta,\phi,\gamma)\ p(\theta,\phi,\gamma).$$

$$\langle n_M \rangle_{Fc} = \frac{1}{4\pi^2} \int_0^{\pi} d\theta \int_0^{2\pi} d\phi \int_0^{\pi} d\gamma \sin\theta \delta_{Fc}(\theta,\phi,\gamma)\; n_M(\theta,\phi,\gamma)\; p(\theta,\phi,\gamma).$$

The above equations are again integrated using Monte Carlo integration, for the parameters given in Table **Error! Reference source not found.**. The results of this calculation is that for truly random binding, the overall ratio of pG to pM binding (which is equal to the ratio of Fc to Fab exposure) would be

$$\frac{\langle n_G \rangle}{\langle n_M \rangle} = \frac{P_{Fab}^0 \langle n_G \rangle_{Fab} + P_{Fc}^0 \langle n_G \rangle_{Fc}}{P_{Fab}^0 \langle n_M \rangle_{Fab} + P_{Fc}^0 \langle n_M \rangle_{Fc}} = 1.05.$$

This ratio is considerably higher than the experimentally observed ratio of about 0.65. We attribute this difference to a small preference for Fc-bound configurations due, possibly, to a higher accessibility of lysine groups there. This preference may be accounted for by assigning adjusted probabilities to Fab- and Fc-bound conjugation instead; we find that using adjusted probabilities

$$P_{Fab} = 0.38, P_{Fc} = 0.62$$

Gives the correct values of 0.65 for the overall ratio of Fc:Fab exposure. The short of this is, that random conjugation is not the same as orientationally averaged binding; the system appears to have a preference for Fc-bound conjugation which results in relatively more pM-binding domain exposure and, generally, more pM bound.

Modeling biased conjugation.

Biasing conjugation is straightforward to implement, as all it does is shift P_{Fab} and thus P_{Fc}. With the above computed quantities, we can compile the data for Fig 5D by computing $\frac{\langle n_G \rangle}{\langle n_M \rangle}$, which is the slope of the lines in Figs 5D and 5E, inserting values of $P_{Fab} = 1, 0.75, 0.5, 0.25$, and 0 in the expression for $\frac{\langle n_G \rangle}{\langle n_M \rangle}$ above.

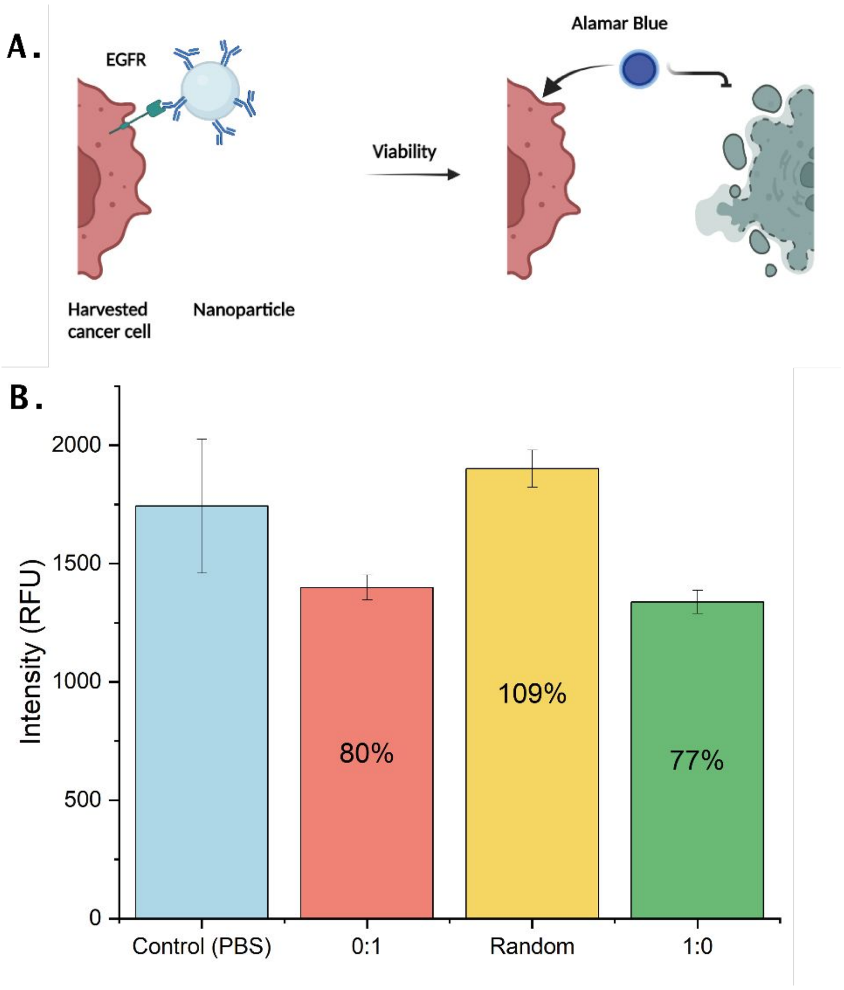

Figure S 13: Viability assay with Alamar Blue. (A) A431 cells were harvested and spiked with the nanoparticles. After incubation, Alamar Blue fluorescence was measured to determine cell death. (B) Percentage of cell survival in the presence of nanoparticles compared to the control with PBS. As can be found, no notable cell death was observed. Schematic was created with Biorender.com

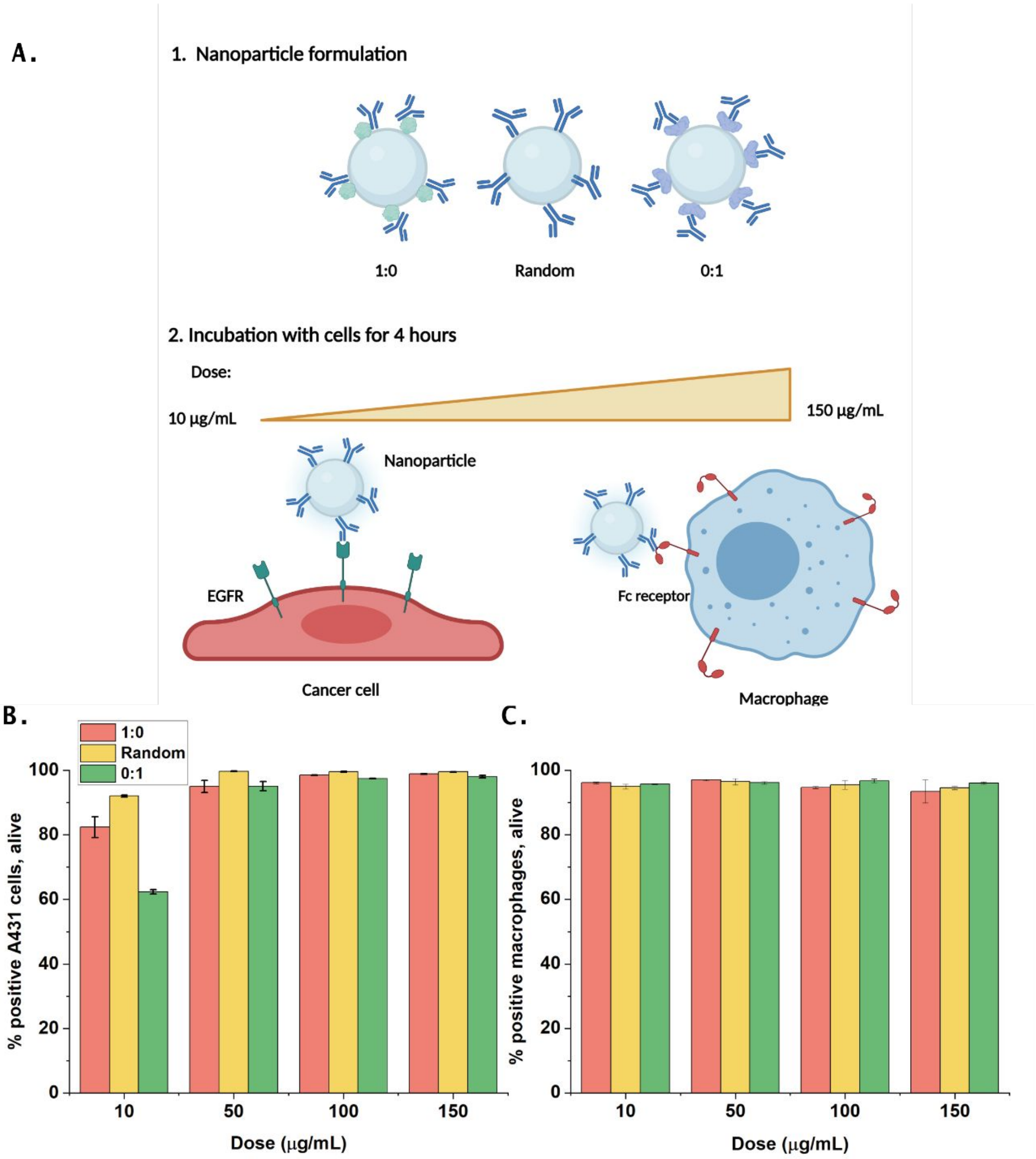

Figure S 14: Nanoparticle bioaccumulation assay with A431 cells and macrophages. A) Schematic overview of the assay. 1) First, the 1:0, random and 0:1 particles were formulated, 2) the nanoparticles were incubated with the two cell types in 4 different dosages; 10 µ/mL, 50 µg/mL, 100 µg/mL and 150 µg/mL for four hours. B) Percentage of positive cells in the part of the A431 population that is alive. D) Percentage of positive cells in the part of the macrophage population that is still alive. Schematic created with Biorender.com.

Table S 4: Overview of the double positive cells that were found in the ADCP assay. For this, the signal in the red channel (eF670) was plotted over the signal in the blue channel (DiO) and the percentage of cells in the top right quadrant were taken.

**Sample**	**Concentration (µg/ml)**	**Double positive cells (%)**	**SD**
COOH bare	10	3.45	0.66
	50	2.62	0.01
	100	2.68	0.91
	150	3.14	1.24
NH_2 bare	10	3.34	1.58
	50	2.90	0.03
	100	3.62	0.36
	150	3.46	0.14
1:0 pG	10	21.60	0.28
	50	23.45	0.21
	100	27.10	1.56
	150	25.45	0.92
Random EDC conjugated	10	16.15	0.07
	50	17.20	0.99
	100	14.40	0.57
	150	15.55	0.21
0:1 pM	10	15.55	1.20
	50	21.90	0.99
	100	26.05	4.17
	150	26.45	0.64

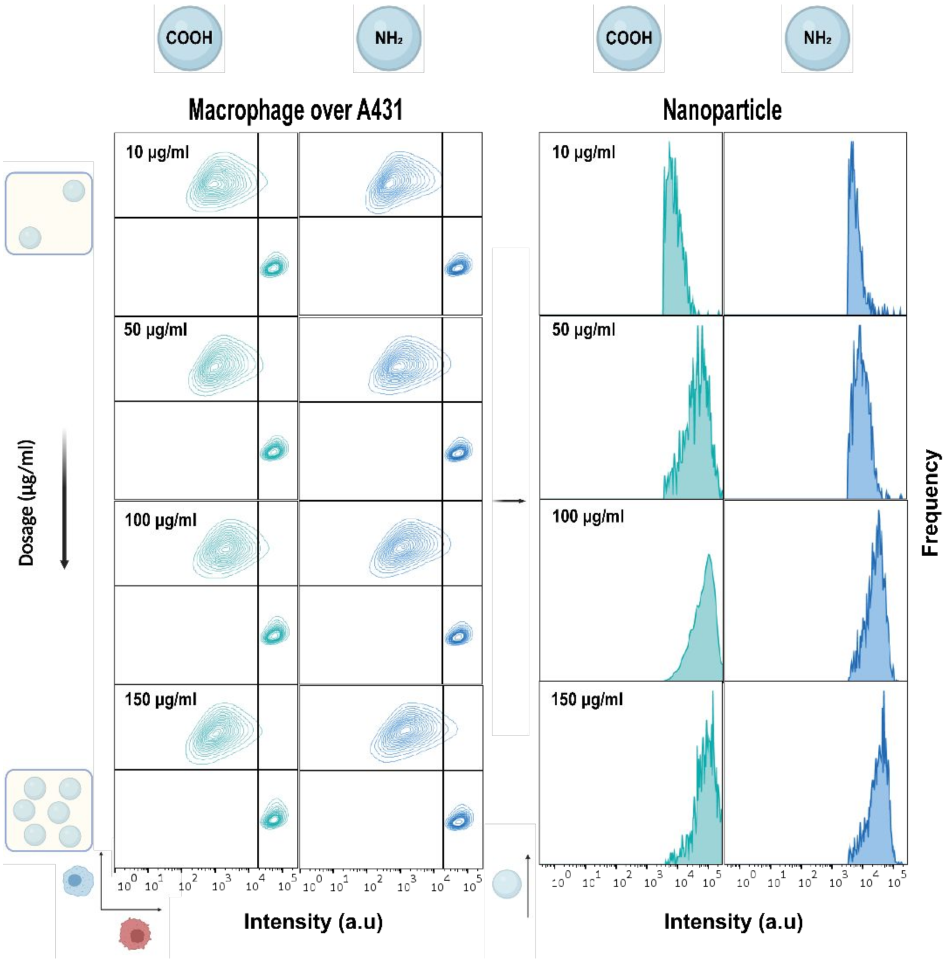

Figure S 15: Flowcytometry results of the negative controls for the ADCP assay. Light blue: COOH bare particles, dark blue: NH_2 bare particles. Left: signal for macrophages plotted over signal for cancer cells at different nanoparticle concentrations. Top right quadrant: double positive cells. These cells were gated and the signal in the nanoparticle channel was analysed (right).

```
atgggctggtcccatccgcagttcgagaaaggtaccatgacatttaaactgataatcaac
 M  G  W  S  H  P  Q  F  E  K  G  T  M  T  F  K  L  I  I  N
ggcaaaaccttaaaaggggagatcacaattgaggcagtcgatgcctaggaagccgagaaa
 G  K  T  L  K  G  E  I  T  I  E  A  V  D  A  -  E  A  E  K
atctttaaacaatatgctaatgattatggtattgacggagaatggacgtatgacgatgcg
 I  F  K  Q  Y  A  N  D  Y  G  I  D  G  E  W  T  Y  D  D  A
acaaaaactttcaccgtaactgaggaattcactagtggtggaagtggggacgatcatcat
 T  K  T  F  T  V  T  E  E  F  T  S  G  G  S  G  D  D  H  H
catcatcatcattgctga
 H  H  H  H  C  -
```

Figure S 16: DNA and amino acid sequence of pG. Areas of interest are indicated as follows: Strep-tag (magenta), protein G (yellow), amber stop codon (bold), His-tag (grey), cysteine site (green)

```
atgggcagcagccatcatcatcatcatcacagcagcggcctggtgccgcgcggcagccat
 M  G  S  S  H  H  H  H  H  H  S  S  G  L  V  P  R  G  S  H
atgtcgcttagtcttaatgacggctcgtatcaaagtgagatcgatctttccggaggtgcg
 M  S  L  S  L  N  D  G  S  Y  Q  S  E  I  D  L  S  G  G  A
aacttccgggagaagtttcgtaatttcgctaatgagctttcagaggctataaccaacagt
 N  F  R  E  K  F  R  N  F  A  N  E  L  S  E  A  I  T  N  S
ccgaagggactggacagacctgtcccaaagacagaaataagcggcctgattaaaacggga
 P  K  G  L  D  R  P  V  P  K  T  E  I  S  G  L  I  K  T  G
gataacttcattacaccgagcttcaaggcgggatactatgatcacgtcgcatctgacggt
 D  N  F  I  T  P  S  F  K  A  G  Y  Y  D  H  V  A  S  D  G
tccttactttcgtattaccaaagtacggagtacttcaataaccgcgtgctgatgcccata
 S  L  L  S  Y  Y  Q  S  T  E  Y  F  N  N  R  V  L  M  P  I
ttgcaaaccacgaatgggacattaatggcgaataaccgcggctacgacgatgtattccgg
 L  Q  T  T  N  G  T  L  M  A  N  N  R  G  Y  D  D  V  F  R
caggtcccatcgttctcgggctggagcaatactaaggccaccactgtatcaacttcgaat
 Q  V  P  S  F  S  G  W  S  N  T  K  A  T  T  V  S  T  S  N
aatcttacctatgacaagtggacgtacttcgccgcaaaaggctcacctctgtatgatagc
 N  L  T  Y  D  K  W  T  Y  F  A  A  K  G  S  P  L  Y  D  S
tacccttgtcatttttttgaagatgtcaaaacattagctatcgatgctaaagatatctcg
 Y  P  C  H  F  F  E  D  V  K  T  L  A  I  D  A  K  D  I  S
gccttaaaaactactatcgattcggagaaacccacatatttgataatacgcggactgtct
 A  L  K  T  T  I  D  S  E  K  P  T  Y  L  I  I  R  G  L  S
ggcaatggctcgcagttgaatgagctgcaattgcccgagtctgtcaaaaaagtctccctt
 G  N  G  S  Q  L  N  E  L  Q  L  P  E  S  V  K  K  V  S  L
tatggagattataccggggttaatgtcgccaaacaaatttttgcgaatgtagttgaactt
 Y  G  D  Y  T  G  V  N  V  A  K  Q  I  F  A  N  V  V  E  L
gaattttacagcacaagcaaggcaaatagtttcggattcaacccgttggtattaggatca
 E  F  Y  S  T  S  K  A  N  S  F  G  F  N  P  L  V  L  G  S
aagaccaatgtaatttatgacttattcgcgtcaaaaccttttactcacatagatttgacc
 K  T  N  V  I  Y  D  L  F  A  S  K  P  F  T  H  I  D  L  T
caggtaaccctgcaaaactccgacaattccgcaatcgatgctaacaaactgaaacaagca
 Q  V  T  L  Q  N  S  D  N  S  A  I  D  A  N  K  L  K  Q  A
gtgggcgacatctataactatagaagatttgaacgccaatttcagggttactttgcagga
 V  G  D  I  Y  N  Y  R  R  F  E  R  Q  F  Q  G  Y  F  A  G
ggctatatcgacaaataccttgtgaaaaatgtcaacactaacaaggactctgacgacgat
 G  Y  I  D  K  Y  L  V  K  N  V  N  T  N  K  D  S  D  D  D
ttagtataccggagtttgaaagagcttaatctgcatttggaagaggcctatcgggaaggc
 L  V  Y  R  S  L  K  E  L  N  L  H  L  E  E  A  Y  R  E  G
gataatacatattaccgcgtaaacgagaactactatccgggtgcatcaatctacgaaaac
 D  N  T  Y  Y  R  V  N  E  N  Y  Y  P  G  A  S  I  Y  E  N
gagcgggcgagccgcgattccgaatttcaaaacgagatccttaaacgtggtggaagtggg
 E  R  A  S  R  D  S  E  F  Q  N  E  I  L  K  R  G  G  S  G
ggcagctggtcccatccgcagttcgagaaatga
 G  S  W  S  H  P  Q  F  E  K  -
```

Figure S 17: DNA and amino acid sequence of pm. Areas of interest are indicated as follows: His-tag (grey), protein M (yellow) and Strep-tag (magenta). In green is the N235C mutation site.

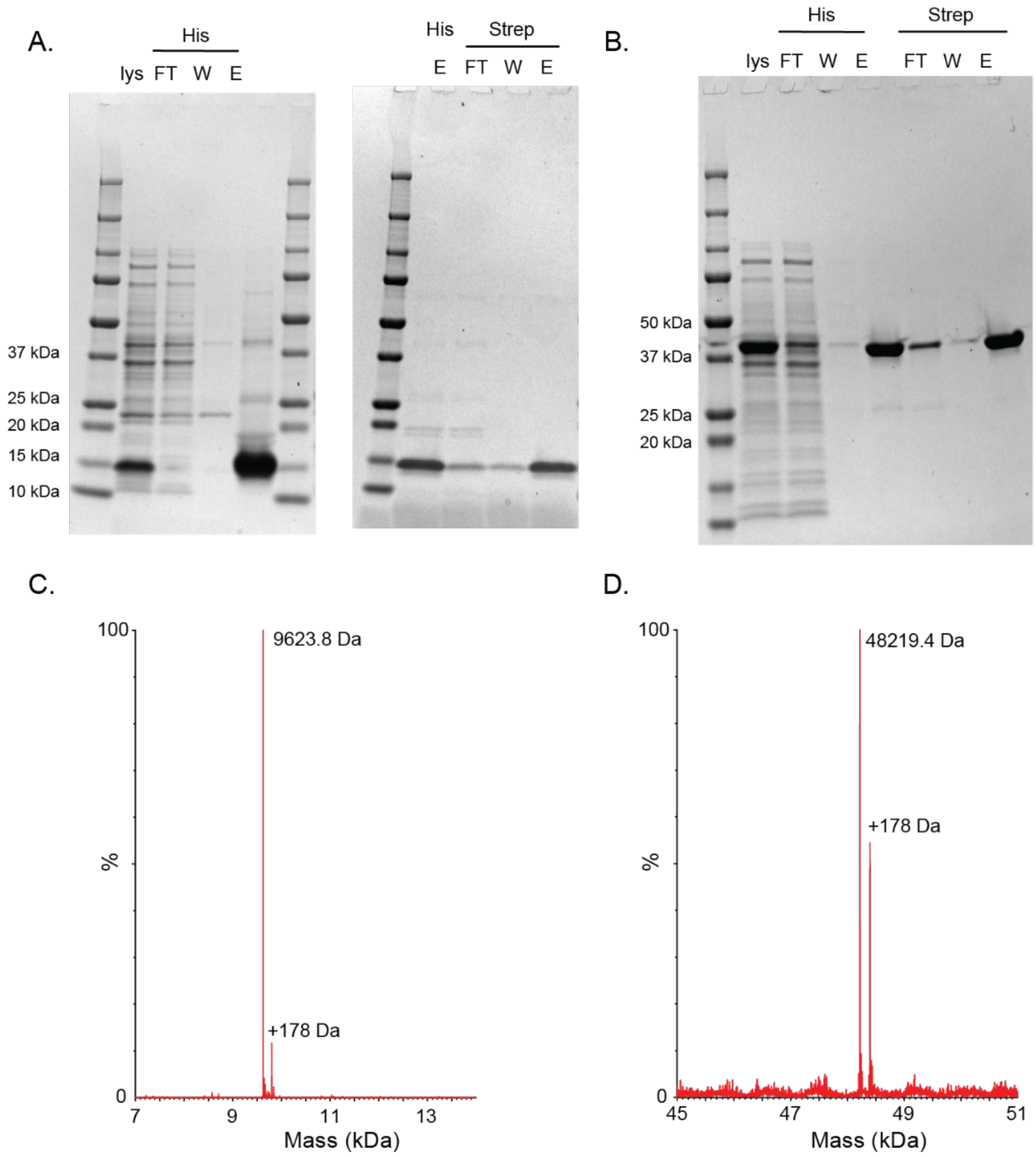

Figure S 18: Purification and characterization of pG and pM. (A-B) SDS-PAGE gel of pG (A) and pM (B) expression and purification. Labels: lys, soluble fraction of cell lysate; His, Ni-affinity chromatography; Strep, Strep-Tactin chromatography; FT, flow through fractions; W, wash fractions; E, elution fractions. (C) Deconvoluted mass spectrum of the elution fractions of pG, showing a single peak at 9623.8 Da corresponding to the calculated mass without N-terminal methionine (9623.4 Da). (D) Deconvoluted mass spectrum of the elution fractions of pM, showing a major peak at 48219.4 Da corresponding to the calculated mass without N-terminal methionine (48219.9 Da). In both cases, the minor peak (+178 Da) corresponds to the intact protein including a formylated N-terminal methionine, commonly observed in prokaryotic protein expression.
